# Development of a Non‐Coding‐RNA‐based EMT/CSC Inhibitory Nanomedicine for In Vivo Treatment and Monitoring of HCC

**DOI:** 10.1002/advs.201801885

**Published:** 2019-03-07

**Authors:** Ruomi Guo, Zhiqiang Wu, Jing Wang, Qingling Li, Shunli Shen, Weiwei Wang, Luyao Zhou, Wei Wang, Zhong Cao, Yu Guo

**Affiliations:** ^1^ Department of General Surgery The First Affiliated Hospital of Sun Yat‐Sen University Guangzhou 510080 China; ^2^ Department of Radiology and VIP Medical Center The Third Affiliated Hospital of Sun Yat‐Sen University Guangzhou 510630 China; ^3^ Department of Radiation Oncology Tianjin Medical University Cancer Institute & Hospital Key Laboratory of Cancer Prevention and Therapy National Clinical Research Center for Cancer Tianjin's Clinical Research Center for Cancer Tianjin 300060 China; ^4^ Department of Obstetrics and Gynecology and Medical Ultrasonics The First Affiliated Hospital of Sun Yat‐Sen University Guangzhou 510080 China; ^5^ School of Biomedical Engineering Sun Yat‐Sen University Guangzhou 510006 China

**Keywords:** cancer stem cells, epithelial–mesenchymal transition, hepatocellular carcinoma, microRNA‐125b‐5p, nanocarriers

## Abstract

The objective of this study is to improve the overall prognosis of patients with hepatocellular carcinoma (HCC); therefore, new therapeutic methods that can be used in vivo are urgently needed. In this study, the relationship between the quantities of microRNA (miR)‐125b‐5p in clinical specimens and clinicopathological parameters is analyzed. A folate‐conjugated nanocarrier is used to transfect miR‐125b‐5p in vivo and to observe the therapeutic effect on HCC. The inhibitory effect and mechanism of miR‐125b‐5p on hepatoma cells are also studied. Data from clinical specimens and in vitro experiments confirm that the miR‐125b‐5p quantity is negatively correlated with progression, and the target protein that regulates the epithelial–mesenchymal transition (EMT)/cancer stem cells (CSC) potential in HCC is STAT3. The miR‐125b‐5p/STAT3 axis inhibits the invasion, migration, and growth of HCC via inactivation of the wnt/β‐Catenin pathway. miR‐125b‐5p‐loaded nanomedicine effectively inhibits the EMT/CSC potential of hepatoma cells in vivo together with their magnetic resonance imaging (MRI) visualization characteristics. An HCC‐therapeutic and MRI‐visible nanomedicine platform that achieves noninvasive treatment effect monitoring and timely individualized treatment course adjustment is developed.

## 1 Introduction

Hepatocellular carcinoma (HCC) is the sixth most commonly diagnosed cancer and the fourth leading cause of cancer death worldwide, primarily due to its high incidence and occurrence of recurrence and metastasis.^1^ Surgical resection, liver transplantation, and local ablation are possibly beneficial only in early stage patients and are still associated with 5‐year mortality rates of 20–40% (resection) and 30–60% (ablation).^2^, ^3^, ^4^ As a result, tumor recurrence is common and endangers the overall survival of HCC patients. Moreover, the treatment of patients with advanced HCC is facing more challenges, and the 5‐year survival rate is even lower. Accordingly, the overall prognosis of HCC after traditional surgical therapies remains disappointing, and inhibition of recurrence and metastasis of HCC via adjuvant treatments has been the focus of clinical research in the last decade. Currently, there is no standard of care for existing adjuvant therapy, because no therapy has a proven benefit in patients with HCC after potentially curative treatment.^5^, ^6^ The antiangiogenic drug sorafenib, which is a representative of adjuvant treatments, has been the most widely studied and most promising treatment in this setting and may provide a better therapeutic effect. However, this drug failed to show any benefit in the adjuvant setting for HCC in a large clinical trial called STROM.^7^

Although most adjuvant treatments have failed to demonstrate efficacy in HCC, a hopeful strategy for healing HCC is to inhibit specific oncogenes using small noncoding RNAs, such as small interfering RNAs (siRNAs) and microRNAs (miRNAs). This approach is currently under investigation in several clinical trials for cancer treatment.^8^ In contrast to siRNAs, which target a specific gene, miRNAs regulate hundreds of mRNA targets simultaneously and accordingly represent an even more appealing tool for cancer therapy.^9^, ^10^ miRNAs have been shown to exhibit impaired expression in various malignant cancers, including HCC, and are involved in HCC recurrence and metastasis through regulation of the epithelial–mesenchymal transition (EMT) and stemness characteristics of hepatoma cells.^11^ Restoration of the expression of tumor suppressor miRNAs normalizes cellular homeostasis and improves therapeutic response of cancer cells.^12^, ^13^ Restricted previous studies showed that microRNA (miR)‐125b‐5p might be associated with clinicopathological features involved in the recurrence and metastasis of HCC and was probably dysregulated in HCC tissues^14^ and patient sera.^15^ Moreover, it was reported that miR‐125b‐5p could inhibit proliferation and metastasis in vitro in breast cancer^16^ and laryngeal cancer.^17^ These findings suggest that miR‐125b‐5p can be used as a candidate drug for HCC treatment following efficient transport into hepatoma cells in vivo.

Despite the low in vivo stability, low cytomembrane permeability, and nonspecific tissue distributions of miRNAs, researchers have studied ideal methods to transport them into target cancer cells with various delivery systems, including nanostructured carriers, and have achieved significant progress.^18^ Our previous study^19^ showed that folate acid (Fa)‐conjugated nanocarrier Fa‐polyethyleneglycol (PEG)‐g‐polyetherimide (PEI)‐superparamagnetic iron oxide nanoparticles (SPION) were an ideal low‐toxicity candidate vehicle for direct gene delivery to HCC cells in vivo. Simultaneous use of the so‐called theranostic strategy to regulate oncogene expression in vivo in an easily trackable manner is possible with noninvasive imaging tools, such as magnetic resonance imaging (MRI). Therefore, Fa‐PEG‐g‐PEI‐SPION (FaPPS) loaded with miR‐125b‐5p could be a potential nanomedicine for inhibiting HCC in vivo.

This study first explored the quantity of miR‐125b‐5p in clinical HCC specimens and its relationship with clinicopathological features. Then, the function and mechanism of miR‐125b‐5p in inhibiting the EMT and cancer stem cells (CSC) of hepatoma cells were studied in vitro. Ultimately, a nanomedicine for in vivo treatment was constructed by loading miR‐125b‐5p onto an Fa‐targeted nanocarrier. The in vivo distribution of nanomedicines was monitored by MRI, and the inhibitory effects of nanomedicines on the EMT and stemness were studied in orthotopic liver HCC and subcutaneous transplantation HCC models.

## 2 Results

### 2.1 miR‐125b‐5p is Downregulated in HCC and Negatively Correlated with Progression

**Figure 1:**
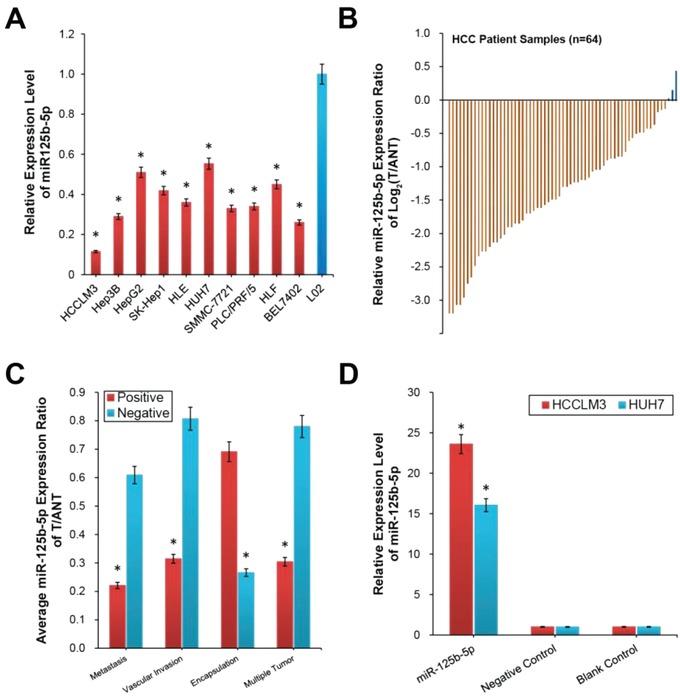
miR‐125b‐5p was downregulated in HCC cell lines and correlated with metastasis‐associated clinicopathological characteristics of HCC patients. A) miR‐125b‐5p expression in ten human HCC cell lines (HCCLM3, Hep3B, HepG2, SK‐Hep1, HLE, HUH7, SMMC‐7721, PLC/PRF/5, HLF, and BEL7402) was lower than that in the hepatic cell line L02 as determined by the RT‐PCR assay. The expression levels were normalized to the L02 expression level. B) RT‐PCR analysis showed that miR‐125b‐5p expression was significantly lower in 64 pairs of HCC tumor (T) tissues than in adjacent nontumor (ANT) tissues. C) Clinicopathological analysis revealed that the average miR‐125b‐5p T/ANT expression ratio of patients was significantly positively correlated with metastasis, vascular invasion, and multiple tumor numbers but was negatively correlated with tumor encapsulation. D) RT‐PCR analysis of miR‐125b‐5p expression in the indicated stable cell lines. The expression levels were normalized to the expression level of the Blank Control cells. Each bar represents the mean ± SD of three independent experiments. Each experiment was repeated three times. (*n* = 3, *P < 0.01).

miR‐125b‐5p expression in various human hepatoma cell lines, including HCCLM3, Hep3B, HepG2, SK‐Hep1, HLE, HUH7, SMMC‐7721, PLC/PRF/5, HLF, and BEL7402, and in a human hepatocyte cell line (L02) was measured by real‐time polymerase chain reaction (RT‐PCR). The results revealed that miR‐125b‐5p was significantly repressed in HCC cells compared to that in L02 cells (**Figure** **1**A). Next, miR‐125b‐5p expression was examined in 64 pairs of HCC tumor (T) and adjacent nontumor (ANT) tissues. The RT‐PCR analysis showed that miR‐125b‐5p expression was significantly lower in the tumor tissues than in the ANT tissues (*P* < 0.01, Figure 1B). Moreover, we compared the average miR‐125b‐5p expression levels in the positive and negative T/ANT groups in terms of metastasis, vascular invasion, encapsulation, and multiple tumor numbers. The average miR‐125b‐5p expression ratio was significantly lower in the positive group than in the negative group for metastasis, vascular invasion, and multiple tumor numbers but was significantly higher for encapsulation (*P* < 0.01, Figure 1C).

### 2.2 miR‐125b‐5p Inhibits HCC Invasion and Migration, the EMT, and Stemness in vitro

**Figure 2:**
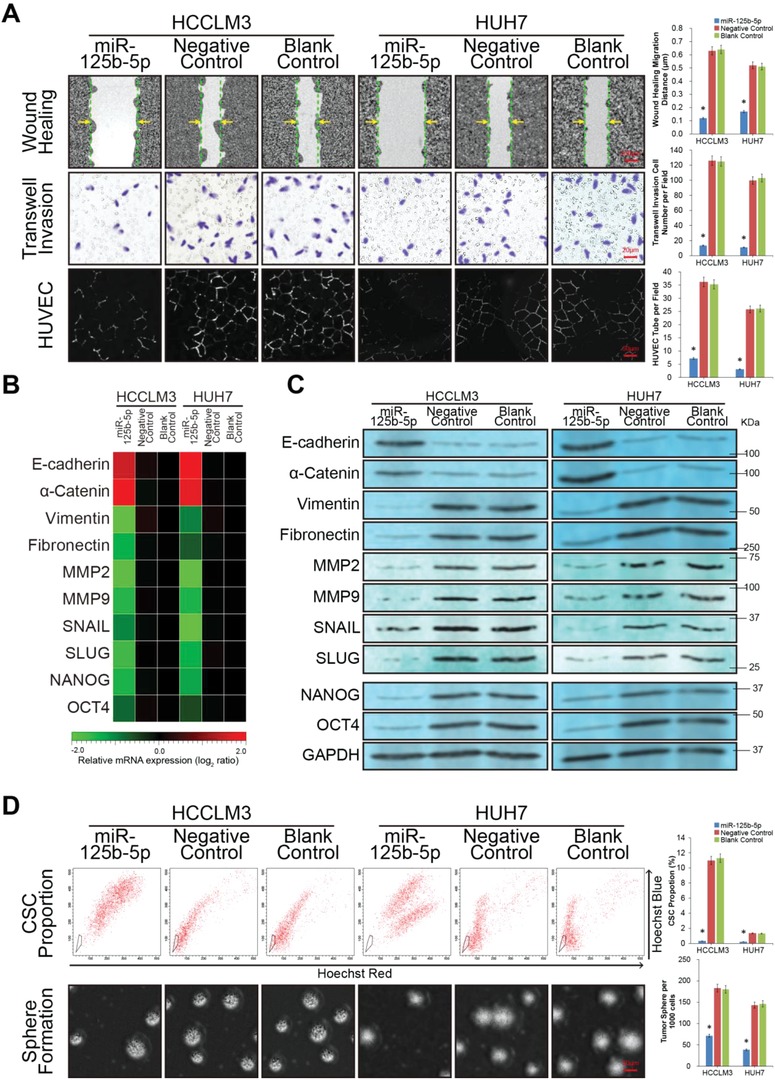
miR‐125b‐5p suppresses the EMT and stemness of HCC in vitro. A) The in vitro migration, invasiveness, and angiogenesis potential of HCC cells were accessed by the wound healing assay, Matrigel‐coated Transwell assay and human umbilical vein endothelial cell (HUVEC) tube formation assay, respectively. The migration, invasiveness, and angiogenesis potential of the miR‐125b‐5p‐overexpressing HCC cells were suppressed compared with those of the control groups. B) The RT‐PCR assay showed the mRNA expression levels of epithelial markers (E‐cadherin and α‐Catenin), mesenchymal markers (Vimentin, Fibronectin, MMP2, MMP9, SNAIL, and SLUG), and cancer stem cells (CSC) markers (NANOG and OCT4) in the indicated groups. The expression levels were normalized to the expression levels of the Blank Control cells. The pseudocolour represents the intensity scale for indicated groups calculated by log_2_ transformation (see also Figure S1A of the Supporting Information). C) Western blotting assay showing the protein expression levels of epithelial–mesenchymal transition (EMT) markers and CSC markers in the indicated groups. The epithelial markers were upregulated, whereas the mesenchymal markers and CSC markers were downregulated in the miR‐125b‐5p group compared with those of the control groups. D) The in vitro proportion and self‐renewal properties of CSC were detected by the flow cytometry side population cell assay (see also Figure S1B,C of the Supporting Information) and tumor sphere formation assay, respectively. The in vitro CSC proportions and sphere volume/count of the miR‐125b‐5p group HCC cells were suppressed compared with those of the control groups. All values are shown as the mean ± SD. Each experiment was repeated three times. (*n* = 3, *P < 0.01).

Considering the above findings, we explored the biological significance and mechanisms of miR‐125b‐5p in HCC tumorigenesis by transducing HCCLM3 and HUH7 cells with retroviral constructs to establish stable miR‐125b‐5p‐overexpressing cell lines. The RT‐PCR analysis showed successful construction of the stable cell lines (*P* < 0.01, Figure 1D). Then, wound healing migration and Matrigel‐coated Transwell invasion assays were performed. The data showed that compared to that of the control cells, miR‐125b‐5p dramatically inhibited the wound closure rate in both the HCCLM3 and Huh7 hepatoma cells (**Figure** **2**A, *P* < 0.01). The Matrigel‐coated Transwell assays demonstrated that miR‐125b‐5p significantly impaired the invasion ability of the HCC cells (Figure 2A, *P* < 0.01). Furthermore, a human umbilical vein endothelial cell (HUVEC) tube formation assay was employed to determine whether miR‐125b‐5p affected tumor angiogenesis. As shown in Figure 2A, conditioned medium from miR‐125b‐5p‐overexpressing hepatoma cells had a weak tube formation induction effect on the HUVECs (*P* < 0.01).

Migration, invasiveness, and angiogenesis functions were reported to be positively correlated with the tumor EMT process.^20^, ^21^ Therefore, we explored the expression levels of EMT markers in each group of hepatoma cells. The RT‐PCR and western blotting assays showed that epithelial markers, including E‐cadherin and α‐Catenin, were upregulated in the miR‐125b‐5p group compared with the levels in the control groups. However, mesenchymal markers, including Vimentin and Fibronectin, were suppressed in the miR‐125b‐5p group hepatoma cells. Moreover, the expression of MMP2, MMP9, SNAIL, and SLUG, which were important for enhancing invasion and promoting EMT program, were all inhibited by miR‐125b‐5p (*P* < 0.01, Figure 2B,C; Figure S1A, Supporting Information).

Previous studies have demonstrated that the EMT process in cancer cells can be promoted by the activity of CSC.^22^, ^23^ Therefore, we examined the effect of miR‐125b‐5p on the stemness of liver cancer cells to investigate the biological mechanisms that inhibit invasion, migration, and angiogenesis. To clarify the functions of miR‐125b‐5p in liver CSC, we examined the percentage of side population cells in hepatoma cells to denote the proportion of CSC.^24^ The proportion of CSC was obviously decreased in the miR‐125b‐5p‐overexpressing HCCLM3 and HUH7 cells, respectively (*P* < 0.01, Figure 2D; Figure S1B,C, Supporting Information). The influence of miR‐125b‐5p overexpression on the self‐renewal property of CSC was investigated using the tumor sphere formation assay. Notably, miR‐125b‐5p‐overexpressing HCCLM3 or HUH7 cells exhibited reduced sphere number and decreased cell content per sphere compared with the spheres formed by the Blank Control cells (*P* < 0.01, Figure 2D). The sphere volume and cell content in the Negative Control groups did not change, because the Negative Control microRNAs lacked an miR‐125b‐5p quantity regulation function (Figure 1D, *P* < 0.01). Furthermore, RT‐PCR and western blotting assay revealed that CSC markers, including NANOG and OCT4, were decreased in the miR‐125b‐5p‐overexpressing hepatoma cells compared with the levels in the Negative and Blank Control cells (*P* < 0.01, Figure 2B and C; Figure S1A, Supporting Information).

### 2.3 miR‐125b‐5p Directly Targets STAT3 in Hepatoma Cells

**Figure 3:**
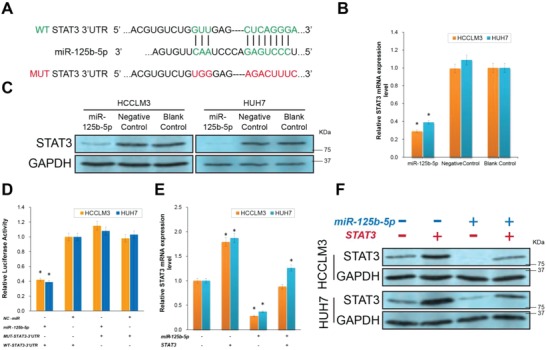
miR‐125b‐5p directly targets STAT3 in hepatoma cells. A) Predicted miR‐125b‐5p direct target sequences in the wild type (WT) 3′‐untranslated region (UTR) of signal transducer and activator of transcription 3 (STAT3) (WT‐STAT3‐3′ UTR) and designed mutant (MUT) nucleotide sequence in the MUT‐STAT3‐3′UTR. B) RT‐PCR assay showing the mRNA expression level of STAT3 in the indicated groups. The expression levels were normalized to the expression level of the Blank Control cells. C) Western blotting assay showing the STAT3 protein expression level. The STAT3 expression level was downregulated in the miR‐125b‐5p group compared with that of the control groups. D) Overexpression of miR‐125b‐5p significantly suppressed the luciferase activity of the WT‐STAT3‐3′UTR in HCC cells, whereas miR‐125b‐5p did not influence the luciferase activity of the MUT‐STAT3‐3′UTR. The expression levels were normalized to the expression level of the (Negative Control) NC‐miR/WT‐STAT3‐3′UTR cotransfected cells. E) RT‐PCR assay showing the STAT3 mRNA expression level in the indicated groups. The expression levels were normalized to the expression level in the miR‐125b‐5p (–)/STAT3 (–) cells. F) Western blotting assay showing the STAT3 protein expression level in the indicated groups. The STAT3 expression level was downregulated by miR‐125b‐5p overexpression but rescued by STAT3 ectopic plasmid transfection. All experiments were performed in triplicate, and the data from each group are presented as the mean ± SD. Each experiment was repeated three times. (*n* = 3, *P < 0.01).

The abovementioned inhibitory effect of miR‐125b‐5p on the EMT and CSC urged us to further explore its therapeutic mechanism in hepatoma cells. miRNAs bind the 3′ untranslated regions (3′UTRs) of mRNAs and inhibit the expression of target proteins.^25^, ^26^ We searched public databases for candidate targets of miR‐125b‐5p. Complementary sequences of miR‐125b‐5p were discovered in the 3′UTR of the signal transducer and activator of transcription 3 (STAT3) mRNA using three most‐used publicly available algorithms, including TargetScan (targetscan.org), PicTar (picta.org), and miRanda (microRNA.org) (**Figure** **3**A). The RT‐PCR and western blotting assays revealed that STAT3 expression was significantly downregulated in the miR‐125b‐5p‐overexpressing cells compared to that in the Negative and Blank Control groups (Figure 3B,C, *P* < 0.01). Consistent with previously available reports, Sirt7^27^, ^28^ and MMP11,^29^ direct targets of miR‐125b‐5p, were also downregulated by miR‐125b‐5p in HCCLM3 and HUH7 cells. However the expression of another reported target, c‐Raf,^30^ did not get affected by miR‐125b‐5p (Figure S2, Supporting Information). To examine whether miR‐125b‐5p directly bound to the STAT3 3′UTR, dual‐luciferase reporter gene assays were performed. As shown in Figure 3D, miR‐125b‐5p overexpression in HCCLM3 and HUH7 cells significantly suppressed the luciferase activity of the wild type (WT) STAT3 3′UTR but not the mutated (MUT)‐STAT3 3′UTR (with miR‐125b‐5p targeting site mutated as shown in Figure 3A) (*P* < 0.01). Finally, a STAT3 eukaryotic expression plasmid rescue experiment was performed to verify the role of miR‐125b‐5p in inhibition of STAT3 expression. The STAT3 and miR‐125b‐5p plasmids were cotransfected into HCCLM3 and HUH7 cells. Then, the RT‐PCR and western blotting assays revealed that the STAT3 mRNA and protein expression levels were significantly downregulated in the miR‐125b‐5p‐overexpressing cells not transfected with the STAT3 plasmid. However, due to WT‐3′UTR independent ectopic STAT3 expression from the STAT3 plasmid, the quantity of STAT3 in the miR‐125b‐5p‐overexpressing cells was significantly reloaded (Figure 3E,F, *P* < 0.01). Taken together, the data from both HCC cell lines suggest that STAT3 is a direct downstream target of miR‐125b‐5p in HCC.

### 2.4 miR‐125b‐5p Suppresses the EMT and CSC of HCC via Targeting STAT3

**Figure 4:**
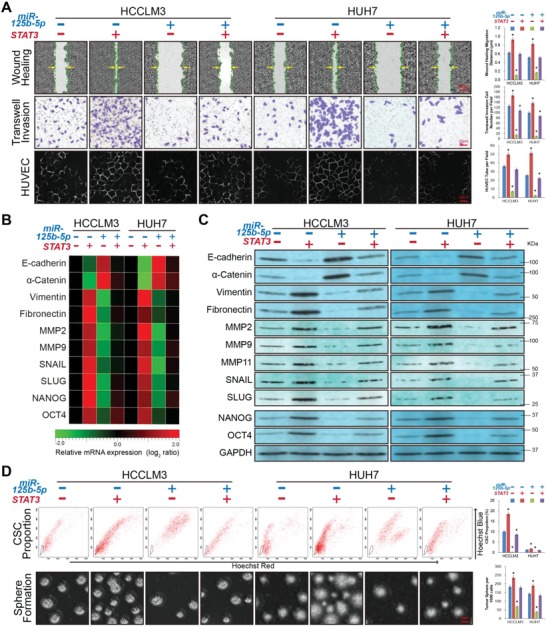
Suppressive effects of miR‐125b‐5p on hepatoma cells depend on direct inhibition of STAT3 and can be antagonized by ectopic STAT3 expression. A) The in vitro migration, invasiveness, and angiogenesis potential of HCC cells was accessed using the wound healing assay, Matrigel‐coated Transwell assay, and HUVEC tube formation assay, respectively, in the indicated groups. The inhibitory effects of miR‐125b‐5p on the migration, invasiveness, and angiogenesis potential were significantly antagonized in the exogenous STAT3 plasmid cotransfected group compared with those of the miR‐125b‐5p‐transfected alone group. B) RT‐PCR assay showing the mRNA expression levels of epithelial markers (E‐cadherin and α‐Catenin), mesenchymal markers (Vimentin, Fibronectin, MMP2, MMP9, SNAIL, and SLUG) and CSC markers (NANOG and OCT4) in the indicated groups. The expression levels were normalized to the expression level of the nontransfected miR‐125b‐5p (‐) / STAT3 (‐) cells (see also Figure S3A of the Supporting Information). The pseudocolour represents the intensity scale for indicated groups, calculated by log_2_ transformation. C) Western blotting assay showing the protein expression levels of EMT markers and CSC markers in the indicated groups. The regulatory effects of miR‐125b‐5p on the EMT and CSC were obviously weakened by the effect of STAT3 ectopic expression. D) The in vitro proportion and self‐renewal property of HCC CSC were detected by the flow cytometry side population cell assay (see also Figure S3B,C of the Supporting Information) and tumor sphere formation assay, respectively. The inhibitory effect of miR‐125b‐5p on stemness was significantly weakened by the increase in STAT3 ectopic expression caused by exogenous plasmid transfection. All values are shown as the mean ± SD of triplicate samples. The experiments were repeated three times. (*n* = 3, *P < 0.01).

Although STAT3 has been shown to be a direct target protein of miR‐125b‐5p in hepatoma cells, its position in the aforementioned therapeutic effect requires a more precise and independent identification process. To verify that the tumor suppressive roles of miR‐125b‐5p in liver cancer were achieved via inhibiting STAT3, we introduced a STAT3 eukaryotic expression plasmid into HCCLM3 and HUH7 cells to induce WT‐STAT3‐3′UTR independent ectopic STAT3 expression. The wound healing assay results demonstrated that reintroducing STAT3 almost entirely reversed the suppressive effect of miR‐125b‐5p in the cotransfected group (**Figure** **4**A, *P* < 0.01). This result was also demonstrated in the Matrigel‐coated Transwell invasion and HUVEC tube formation assays, because rescue of STAT3 expression led to an almost complete reversion of the number of invaded cells and tubular structures in the HUVECs in the miR‐125b‐5p and STAT3 cotransfected group (Figure 4A, *P* < 0.01). EMT‐related markers were analyzed by RT‐PCR assay and western blotting assay, which revealed that E‐cadherin and α‐Catenin were reloaded in the miR‐125b‐5p‐overexpressing cells in a manner that was reversed by STAT3 overexpression. Additionally, miR‐125b‐5p suppressed Vimentin, Fibronectin, MMP2, MMP9, SNAIL, and SLUG expression in the HCCLM3 and HUH7 cells when STAT3 expression was inhibited but not when STAT3 expression was resumed in the cotransfected group (*P* < 0.01, Figure 4B,C; Figure S3A, Supporting Information). Furthermore, rescue of STAT3 expression in the miR‐125b‐5p cotransfected cells recovered virtually all of the CSC, as indicated by the resu med proportion and tumor sphere formation potency (Figure 4D; Figure S3B,C, Supporting Information). Reload of the stemness‐associated markers NANOG and OCT4 was also verified in the miR‐125b‐5p/STAT3 eukaryotic expression plasmid cotransfected group (Figure 4B,C, *P* < 0.01).

Briefly, since ectopic STAT3 protein expression almost entirely antagonized the HCC suppressive effect of miR‐125b‐5p, then miR‐125b‐5p was definitely proven to suppress the EMT and CSC in vitro in a manner that depended on direct targeting of STAT3. The miR‐125b‐5p/STAT3 axis regulates the EMT and CSC potential in HCC cells, which may become a therapeutic target for HCC.

### 2.5 miR‐125b‐5p/STAT3 Axis Regulates the wnt/β‐catenin Pathway in Hepatoma Cells

**Figure 5:**
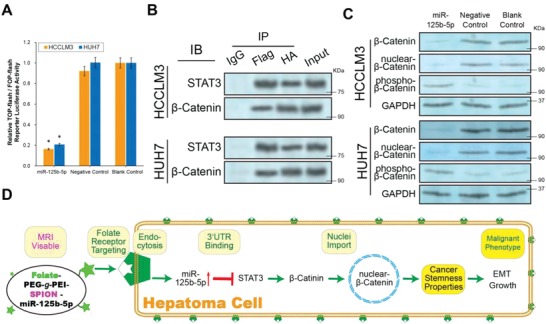
miR‐125b‐5p/STAT3 axis affects the wnt/β‐catenin pathway in hepatoma cells. A) The TOP/FOP flash dual luciferase reporter assay was performed to investigate the potential regulatory effects of miR‐125b‐5p on the transcriptional activity of β‐catenin. Luciferase activity was measured 48 h after transfection and normalized. The luciferase activity of the Blank Control cells was set as 1.0. Overexpression of miR‐125b‐5p eventually suppressed the luciferase activity of β‐Catenin in HCC cells. All experiments were performed in triplicate, and the data from each group are presented as the mean ± SD. (*n* = 3, *P < 0.01). B) HCC cells were transfected with the flag‐STAT3 and HA‐β‐catenin vectors to investigate the protein interactions between STAT3 and β‐catenin. Immunoprecipitation assay confirming the interaction between STAT3 and β‐catenin. C) Western blotting assay showing the total β‐Catenin, nuclear‐β‐Catenin, and phospho‐β‐Catenin protein expression levels. Total β‐Catenin and nuclear‐β‐Catenin were downregulated, whereas phospho‐β‐Catenin was upregulated by reduction of STAT3 in the miR‐125b‐5p group. D) A schematic diagram of the process by which the MRI‐visible nanomedicine identifies the folate receptor internalized by HCC cells and then produces an HCC‐specific therapeutic effect.

The interesting conclusions described above allowed us to develop a drug that suppressed the EMT and CSC through the miR‐125b‐5p/STAT3 axis for HCC treatment in vivo. However, first we needed to clarify the potential signaling pathways through which the miR‐125b‐5p/STAT3 axis regulated the EMT and CSC in hepatoma cells. STAT3 has been reported to have different effects on the wnt/β–Catenin signaling pathway in various types of human cancers, but its relations with HCC is unclear.^31^ Only one preliminary study reported that STAT3 might regulate β‐Catenin protein expression in HCC; however, that study was limited to suggesting relative changes in protein quantities in vitro and did not suggest definitive conclusions and mechanisms.^32^ To further investigate the potential regulatory effects of the miR‐125b‐5p/STAT3 axis on the transcriptional activity of the wnt/β‐Catenin pathway, we performed a TOP/FOP dual‐luciferase reporter assay. The luciferase experiments showed that the wnt/β‐Catenin pathway activity was significantly lower in the miR‐125b‐5p group than that in the control groups (**Figure** **5**A, *P* < 0.01). This result indicated that miR‐125b‐5p influenced wnt/β‐Catenin signaling pathway activity through several steps. Then, we conducted an immunoprecipitation assay (IP) to investigate whether STAT3 was the key protein through which miR‐125b‐5p controlled β‐Catenin activity. The results showed that the STAT3 interacts with the β‐Catenin in HCCLM3 and HUH7 cells (Figure 5B). The western blotting experiments showed that when the STAT3 content was decreased in the miR‐125b‐5p group, total β‐Catenin and nuclear β‐Catenin expression were decreased significantly but the phosphorylated β‐Catenin was increased. Thus, when intracellular STAT3 was reduced, more β‐Catenin was phosphorylated and then subjected to proteolysis. Subsequently, the total intracellular β‐Catenin content decreased and the amount of β‐Catenin that could enter the nucleus decreased significantly, resulting in an eventual decrease in wnt/β‐Catenin signal pathway activity (Figure 5C). After clarifying the therapeutic effect and mechanism of miR‐125b‐5p in HCC, we used the Fa‐PEG‐*g*‐PEI‐SPION (FaPPS) nucleic acid nanocarrier to deliver this microRNA into hepatoma cells to assess the in vivo therapeutic effect. We hypothesized that the Fa‐targeted nanocarrier would help miR‐125b‐5p enter hepatoma cells and inhibit the potential of the EMT and CSC in vivo (Figure 5D).

### 2.6 Effective Recognition, Visualization, and Gene Transmission for Hepatoma Nodes Via Theranostic Nanomedicine

Since the folate‐conjugated nanomedicines used in this study contained the MRI T2 contrast agent SPIONs, visualizing the distribution process with noninvasive MRI scans was easy both in vitro and in vivo. The MRI visible nanocarrier FaPPS can intelligently recognize the folate receptor of hepatoma cells. Recognition of hepatoma cells is achieved by folate conjugated on its surface. We hoped to demonstrate that the specific in vivo distribution of miR‐125b‐5p into HCC cells could be protected by this nanocarrier prior to the folate receptor‐mediated endocytosis process (Figure 5D).

**Figure 6:**
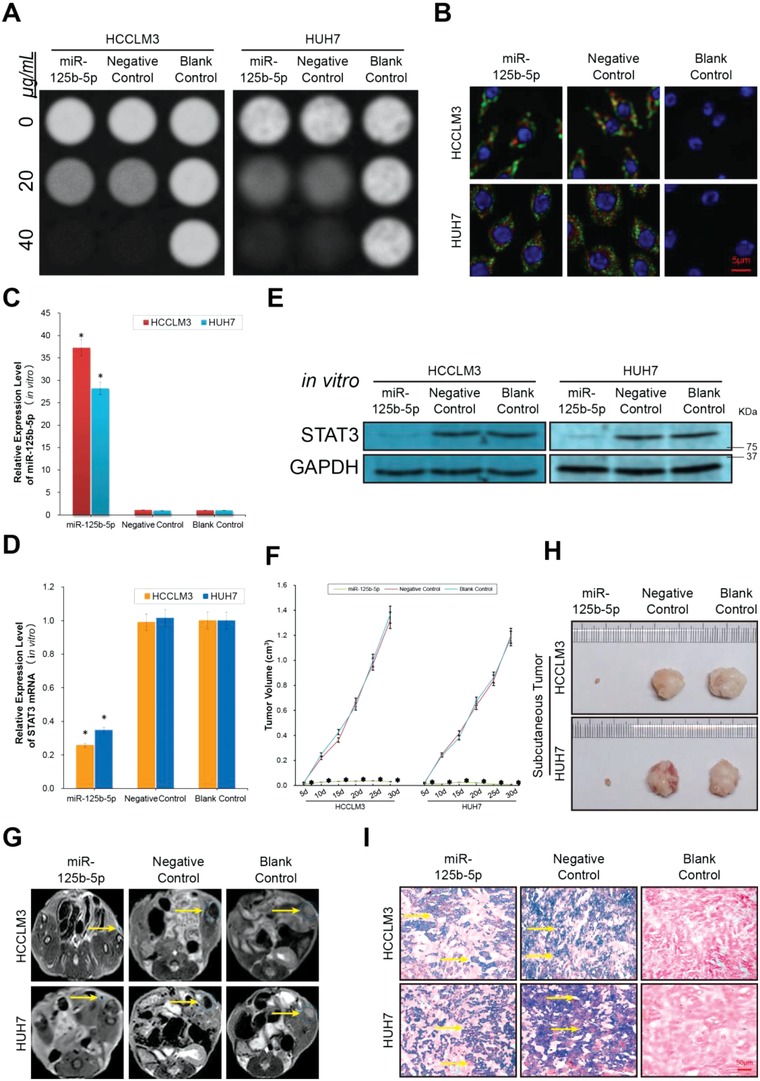
Folate‐conjugated, SPION‐containing, and miR‐125b‐5p‐loaded nanomedicines can simultaneously achieve intelligent recognition, MRI imaging, and gene regulation of hepatoma cells in vivo. A) After 1 h of incubation with various Fe dosages of superparamagnetic iron oxide nanoparticle (SPION)‐containing nanomedicines, the MRI T2 signal intensity of labeled HCCLM3 and HUH7 cells was reduced as the Fe concentration increased. The nanomedicines contain the sensitive MRI contrast agent SPION, which is the reason for the hypointense MRI T2 signal. B) In situ laser confocal microscopy fluorescence images indicating endocytosis of nanomedicines into hepatoma cells. The nanomedicines were provided in the culture medium with an Fe concentration of 20 µg mL^−1^. These results confirmed that the miR‐125b‐5p or Negative Control miR plasmids (red) were transported successfully into the cytoplasm of the HCCLM3 and HUH7 cells accompanied by the folate‐conjugated MRI‐visible nanocarrier (green). (Blue, 4′,6‐diamidino‐2‐phenylindole (DAPI) indicating nuclei; red, POPO‐3 indicating plasmid; green, Oregon Green 488 indicating nanocarrier). C) The miR‐125b‐5p expression level in HCC cells after treatment with nanomedicines containing miR‐125b‐5p or Negative Control miR plasmids detected by the RT‐PCR assay. D) The STAT3 mRNA expression level in HCC cells detected after treatment with nanomedicines containing different plasmids. E) The STAT3 protein expression level after treatment with different nanomedicine detected by western blotting assay. The miR‐125b‐5p‐loaded nanomedicine can improve the quantity of miR‐125b‐5p in hepatoma cells and thus effectively inhibit the expression of the downstream target protein STAT3. F) The therapeutic effect of nanomedicines on tumor growth was observed in the HCCLM3 and HUH7 subcutaneous HCC models. The volumes of the subcutaneous tumors were measured after treatment, and tumor growth curves were obtained to represent the tumor growth rates in the different groups. G) Representative images obtained by MRI T2 scanning after injection of nanomedicines containing different plasmids and SPION on day 30. Compared with that of the Blank Control group injected with PBS, the tumor regions of the nanomedicine treatment groups (miR‐125b‐5p and NC groups) were detected as strongly hypointense areas in the T2‐weighted images. Compared with those of the Negative Control and Blank Control group, the tumor size of the miR‐125b‐5p group was intuitively reduced. The yellow arrows indicate the location of the tumor regions. H) At the end of the treatments, the subcutaneous tumors of the indicated groups were harvested, and the tumor sizes were measured directly. The tumor size data obtained by direct observation support the tumor growth curve and MRI detection results. I) After injection with different nanomedicines or PBS, subcutaneous tumor nodules were harvested for pathological study. Prussian blue iron staining was used to observe the amount of Fe internalized into the in vivo HCC cells with the SPION‐containing nanomedicines. The blue staining indicates that the iron in the SPION has been stained and positioned. The trend in the Fe content in HCC cells was consistent with the trend of the MRI T2 scan signals. The yellow arrows indicate the location of the blue staining regions. Data are representative of 3 (in vitro) or 12 (in vivo) independent experiments as the mean ± SD, and statistical significance was determined as *P < 0.01.

First, in vitro MRI scanning was used to verify the ability of the nanocarrier to recognize hepatoma cells and promote endocytosis while carrying different plasmids. Plasmids containing miR‐125b‐5p or a Negative Control (NC) miR were loaded onto the FaPPS nanocarrier and transfected into HCC cells in the miR‐125b‐5p or NC groups, respectively. The nanocarrier concentration was quantified by the concentration of the contained iron. Hepatoma cells treated with phosphate buffer saline (PBS) were used as the Blank Control group. Compared with that of the Blank Control group, the MRI T2 signal intensities of the HCCLM3 and HUH7 cells decreased, as expected, after 1 h of culture with the SPION‐containing nanomedicine accompanied by an increase in the Fe concentration of the nanomedicine in the miR‐125b‐5p and NC groups. The MRI T2 signal intensity at the 40 µg Fe mL^−1^ concentration was lower than those at the 20 and 0 µg Fe mL^−1^ concentrations. No signal change was observed in the Blank Control group due to the absence of transfection with SPION‐containing nanocarriers (**Figure** **6**A). Plasmids are not visible in the MRI scan, and thus the MRI can only trace the distribution of nanocarriers. We used a fluorescence staining assay to observe whether the plasmids could endocytose into hepatoma cells with the nanocarrier. After culture with the nanomedicine for 1 h, in situ laser confocal microscopy analysis highlighted red (plasmid) and green (nanocarrier) fluorescence in the HCCLM3 and HUH7 cells in the miR‐125b‐5p and NC groups but not in the Blank Control group. These results confirmed that the miR‐125b‐5p and NC miR plasmids were transported successfully into the cytoplasm of HCCLM3 and HUH7 cells accompanied by the folate‐conjugated nanocarrier (Figure 6B). These in vitro appearances demonstrated that FaPPS basal nanomedicines could bind and induce endocytosis into hepatoma cells regardless of the type of nucleic acids they synthesized (Figure 6A,B). The transfection efficiency of the plasmids was detected after nanocarrier‐mediated internalization. The RT‐PCR results demonstrated that the relative miR‐125b‐5p expression level was significantly higher in the miR‐125b‐5p group HCCLM3 and HUH7 cells than in the Negative and Blank Control groups (*P* < 0.01, Figure 6C). The RT‐PCR and western blotting results revealed that the STAT3 expression level was significantly downregulated in the miR‐125b‐5p‐overexpressing nanomedicine‐transfected cells compared with that of the control groups (Figure 6D,E). These performances indicate that after internalization into tumor cells, the ultimate therapeutic effect of the nanomedicine depends on the function of the loaded nucleic acids.

After confirming that nanomedicines have a high targeting distribution function and that the miR‐125b‐5p/STAT3 axis has a regulatory effect in vitro, we introduced a subcutaneous transplantation HCC model to investigate whether nanomedicines could achieve a therapeutic effect in vivo. First, the tumor volume data showed that after miR‐125b‐5p nanomedicine treatment, the growth rate of the subcutaneous tumor was significantly blocked compared with that of the control groups regardless of whether the tumors originated from HCCLM3 or HUH7 cells (Figure 6F, *P* < 0.01). To observe the in vivo duration time of nanomedicines, we checked the concentration of fluorescent labeled FaPPS/miR‐125b‐5p and FaPPS/NC miR from blood after single tail‐vein injection, by determining the fluorescence intensity of the blood at different postinjection time points. The results indicate that the in vivo distribution patterns of the two nanomedicines are similar. For instance, at the time point of 6 h, nearly 50% of the nanomedicines were cleared from blood. Moreover, only less than 5% of the nanomedicines were remained after 24 h (Figure S4, Supporting Information). Additionally, we performed an MRI T2 scan after single tail‐vein injection of nanomedicines and compared the results with those of the Blank Control group injected with PBS. The tumor regions of the nanomedicine treatment groups were detected as strongly hypointense areas in the T2‐weighted images. The imaging results confirmed that regardless of which plasmid was involved, the SPION‐containing nanomedicines achieved an effective in vivo distribution of hepatoma cells (Figure 6G). At the same time, real‐time and noninvasive monitoring was achieved for the treatment response and volume changes in the tumor nodules during in vivo treatment. If nanomedicines, such as the NC group, do not exhibit antitumor effects during the treatment course, this technology can guide doctors to change the therapeutic nucleic acids contained in the nanomedicines in a timely manner.

Next, we harvested the subcutaneous tumor nodules for direct observation and pathological examination. The tumor size data obtained by direct measurement (Figure 6H) were consistent with the tumor growth curve (Figure 6F) and the results obtained from the MRI tumor imaging experiments (Figure 6G). Furthermore, to directly verify whether the MRI signal changes reflected the aggregation of Fe‐containing nanomedicines in the tumor tissue, we performed a Prussian blue staining experiment on the tissue sections. The blue staining of the tumor cells was significantly stronger in the two nanomedicine treatment groups than in the Blank Control group. The results indicated that the distribution of SPION‐containing nanomedicines was positively correlated with the trend in the MRI T2 signal (Figure 6I). Nevertheless, compared with that of the Blank Control group, the NC miR‐containing nanomedicine increased the iron content and decreased the MRI T2 signal in vivo but did not produce a therapeutic effect in terms of inhibition of tumor growth. This finding indicated that after effective distribution of the tumor in vivo, the therapeutic effect of the nanomedicine was determined by the loaded therapeutic genes.

### 2.7 In vivo HCC Therapeutic Effect and Mechanism of miR‐125b‐5p‐loaded Nanomedicine

**Figure 7:**
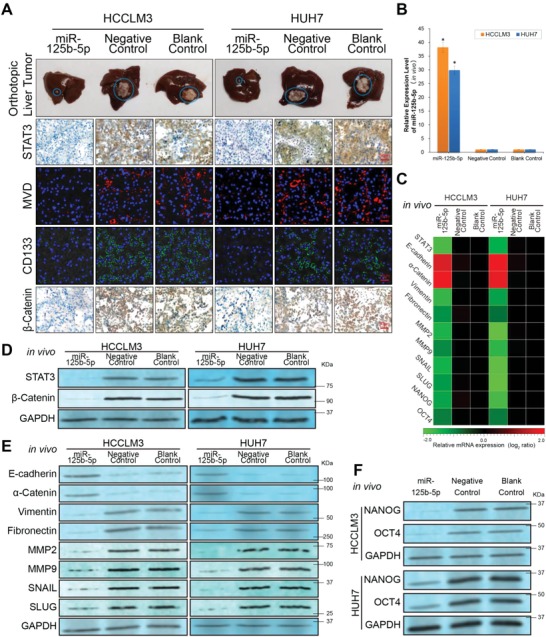
Inhibitory effect and biological mechanisms of miR‐125b‐5p‐loaded nanomedicines on hepatoma EMT/CSC were verified using an orthotopic liver tumor model. A) 1st panel, representative optical imaging of the liver showing orthotopic tumors after treatment in the different groups; 2nd panel, quantity of STAT3 protein determined by the immunohistochemistry (IHC) assay; 3rd and 4th panels, in vivo HCC angiogenesis abilities or CSC ratios measured by the CD34 microvessel density (MVD) staining assay or CD133 immunofluorescence staining assay (Blue, DAPI indicating nuclei; red, CY5 indicating CD34 protein; green, FITC indicating CD133 protein); 5th panel, quantity of β‐Catenin protein determined by the IHC assay. B) The miR‐125b‐5p expression levels in liver orthotopic tumor tissues detected by the RT‐PCR assay. C) Relative mRNA quantities of STAT3, epithelial markers (E‐cadherin and α‐Catenin), mesenchymal markers (Vimentin, Fibronectin, MMP2, MMP9, SNAIL, and SLUG) and CSC markers (NANOG and OCT4) in liver orthotopic tumor tissues were detected by the RT‐PCR assay. The expression levels were normalized to the expression levels of the Blank Control tissues. The pseudocolour represents the intensity scale for indicated groups, calculated by log_2_ transformation. D) The STAT3 and β‐Catenin protein expression levels in the indicated groups of tumor tissues were detected by western blotting assay. The results showed that the STAT3 and β‐Catenin expression levels in the HCC tissues decreased significantly together with the increase in miR‐125b‐5p after treatment with the miR‐125b‐5p‐loaded nanomedicine therapy. E) Western blotting assay showing the protein expression levels of EMT markers in tumor tissues. F) The protein expression levels of CSC markers in HCC tissues were detected by the western blotting assay. The results showed that because of the changes in the miR‐125b‐5p/STAT3 axis caused by the miR‐125b‐5p‐loaded nanomedicine therapy, the in vivo EMT process and stemness property of the in vivo HCC cells were inhibited significantly. The expression levels were normalized to the expression level of Blank Control tissues. The data are representative of 12 independent experiments as the mean ± SD, and statistical significance was determined as *P < 0.01.

Although a subcutaneous tumor model can directly show the in vivo inhibitory effect of nanomedicines on tumor, a liver orthotopic model can better mimic the growth environment and blood supply of HCC nodules. To investigate the in vivo therapeutic effect and mechanism of miR‐125b‐5p‐loaded nanomedicine, we established a liver orthotopic tumor model. As shown in **Figure** **7**A, after nanomedicine treatment, the orthotopic tumor in the miR‐125b‐5p group was significantly smaller than that in the control groups. The results were consistent with those from studies of subcutaneous transplanted tumor models. To investigate whether the inhibitory effect of nanomedicines on in vivo tumors was caused by regulation of the miR‐125b‐5p/STAT3 axis, the miR‐125b‐5p and STAT3 expression levels were detected by RT‐PCR, immunohistochemistry (IHC), and western blotting assays. After treatment with the miR‐125b‐5p‐loaded nanomedicine, the relative miR‐125b‐5p expression level was significantly elevated but the STAT3 expression level was decreased in the HCCLM3 and HUH7 tumors compared to that of the control groups (*P* < 0.01, Figure 7A–D).

In agreement with the in vitro angiogenesis study, the hepatoma angiogenesis potential detected by the microvessel density (MVD) assay also significantly decreased with the increase in the miR‐125b‐5p quantity (Figure 7A). Studies of closely related EMT markers have shown that the EMT process in vivo is also significantly inhibited by miR‐125b‐5p‐loaded nanomedicine therapy. The determination of EMT marker proteins closely related to angiogenesis showed that the EMT process in vivo was significantly affected by the changes in the miR‐125b‐5p/STAT3 axis according to nanomedicine treatment. After treatment with the miR‐125b‐5p‐loaded nanomedicine, the epithelial markers E‐cadherin and α‐Catenin were upregulated but the mesenchymal markers Vimentin, Fibronectin, MMP2, MMP9, SNAIL, and SLUG were downregulated in the HCCLM3 and HUH7 tumor tissues, as determined by RT‐PCR and western blotting assays (Figure 7C,E, *P* < 0.01). This finding proved that the EMT process in vivo was almost completely blocked by the miR‐125b‐5p‐loaded nanomedicine compared with that in the control groups.

In vivo tumor growth and the EMT process are likely regulated by cancer stem cells. To clarify the mechanism underlying the above mentioned in vivo inhibitory effects on HCC, we conducted a series of CSC detection assays. The immunofluorescence assay of tumor slices showed that the proportion of CD133‐positive HCC cells in the tumor tissues was remarkably negatively correlated with the quantity of miR‐125b‐5p (Figure 7A). CD133 is a proven marker of HCC stem cells. CD133^+^ HCC cells were confirmed to have strong tumor angiogenesis ability and were closely related to malignant phenotypes, such as invasion, metastasis, and drug resistance in vivo. The expression levels of CSC markers (NANOG and OCT4) further confirmed the changes in HCC stemness caused by nanomedicine therapy. The expression levels of CSC markers were reduced significantly, which was accompanied by an increase in the miR‐125b‐5p quantity in HCC tissues (Figure 7C,F, *P* < 0.01).

In previous in vitro experiments (Figure 5), stemness was found to be regulated by the wnt/β–Catenin pathway in miR‐125b‐5p group hepatoma cells. Therefore, after discovering that the EMT and stemness of hepatoma cells in vivo were inhibited by the miR‐125b‐5p‐loaded nanomedicine, we evaluated the β‐Catenin protein quantity in the tumor tissues. The results showed that the quantity of β‐Catenin was decreased in the tumor tissues of the miR‐125b‐5p group compared with that of the control groups (Figure 7A,D).

In summary, we monitored the in vivo effective distribution of miR‐125b‐5p‐loaded nanomedicine in subcutaneous tumor tissues by MRI T2 scanning. Furthermore, in an orthotopic liver tumor model, we demonstrated that this nanomedicine remarkably increased the miR‐125b‐5p quantity in the tumor tissues, which in turn inhibited the growth, EMT, and stemness of HCC cells in vivo. Our findings were also supported by the detection of EMT and CSC markers. The inhibition of the EMT and stemness by the miR‐125b‐5p‐loaded nanomedicine may be due to inhibition of STAT3 expression, resulting in a decrease in wnt/β‐Catenin pathway activity. In HCC cells, when the quantity of STAT3 is decreased, the phosphorylation and hydrolysis of β‐Catenin are intensified; thus, entry of β‐Catenin into the nucleus is decreased, and the wnt/β‐Catenin pathway activity eventually decreases.

## 3 Discussion

Despite significant advances in cancer diagnostics and treatment, HCC leads to the deaths of more than 781 631 people every year worldwide.^1^ Currently, treatment for advanced HCC mainly depends on the synergistic effect of combining surgical and adjuvant therapy. Conventional treatments for HCC patients, such as liver resection, transplantation, and chemotherapy, have shown limited efficacy due to the high metastatic and growth rates. As a result of the relatively asymptomatic nature, the advanced stage at the time of diagnosis and the indefinite pathogenic mechanisms, HCC is the most common digestive malignancy worldwide despite the development of various therapeutic strategies. Accordingly, an urgent and essential aim in the struggle with HCC is to achieve a better understanding of the mechanisms responsible for the disease and to develop relevant and more effective treatments. The cancer stem cell model has provided new insights into the growth and metastasis of HCC. CSC are cancer cells that are capable of self‐renewal and have a long‐term repopulation property. Because they are important intermediaries of cancer proliferation, invasion, metastasis, drug resistance, and recurrence, their suppression may lead to improvement of treatment outcomes and patient survival. Over the last decade, the role of the EMT in the invasion and metastasis of HCC has gained broader attention. This multistep versatile procedure has led to a genotype interchange from an epithelial to a mesenchymal cellular state. The EMT has been confirmed to be closely related to activation of CSC in tumor tissues. Additionally, CSC are plastic and are associated with both epithelial state and mesenchymal state. Consequently, exploring the molecular switches that control EMT evolution and CSC function is crucial for developing clinically applicable anti‐HCC medicines.

In this study, clinical specimens were analyzed and in vitro experiments were performed to confirm miR‐125b‐5p as a possible gene therapy target for HCC by inhibiting both the EMT and CSC. Current research on the status of miR‐125b‐5p in HCC is very limited. Using microarray detection of seven pairs of HCC and matched nontumor tissues, Zhu et al found that miR‐125b‐5p might be expressed at a low level in HCC.^25^ Shimagaki et al. analyzed 694 clinical specimens and proved that a low quantity of miR‐125b‐5p in tumor specimens was associated with elevated early recurrence and reduced overall survival of patients after curative HCC resection.^14^ Conversely, Giray et al found that the serum miR‐125b‐5p level was higher in 20 HCC patients than in normal subjects.^15^ However, these studies did not explore the mechanism of miR‐125b‐5p quantity changes, and the limited preliminary studies are not sufficient to explain the role of miR‐125b‐5p in HCC.

Previous studies have found that the actual role of miR‐125b‐5p in different tumor cells may depend on various target proteins. For example, miR‐125b‐5p inhibits breast cancer by targeting KIAA1522,^16^ suppresses esophageal squamous cell carcinoma by regulating HMGA2,^26^ enhances the chemotherapy sensitivity of gallbladder cancer by downregulating Bcl2,^33^ and selectively targets IRF4 in multiple myeloma cells.^34^ In colorectal cancer, it was even reported that CXCL12/CXCR4 axis induced miR‐125b promoted EMT.^35^ However, in this study, we did not notice the coexpression of miR‐125b with CXCL12/CXCR4 axis in HCC. Although some studies have suggested that miR‐125b‐5p is associated with Ets1 in hepatoblastoma,^36^ as far as it is known, its target protein in HCC has not been reported. Further clarification of the molecular biological changes induced by miR‐125b‐5p in hepatoma cells is very important to confirm its inhibitory effects on HCC and develop related therapeutic methods. We predicted that STAT3 might be the target gene of miR‐125b‐5p through bioinformatics analysis. Then, a series of experiments confirmed that STAT3 was the key target protein in the process by which miR‐125b‐5p inhibited the growth, EMT, and CSC of HCC cells and that activation of the miR‐125b‐5p/STAT3 axis effectively reversed the EMT process and inhibited the stemness potential. In addition to STAT3, Smad4 is also predicted as a nonconservative target of miR‐125b‐5p and had been reported associating with EMT and CSC in HCC previously.^37^ Indeed, we did notice that miR‐125b‐5p inhibited Smad4 expression in HUH7 cells but not in HCCLM3 cells. And restoration of the expression of Smad4 rescued the inhibitory roles of miR‐125b‐5p to a much lower level compared with restoration of STAT3 (data not shown). Hence, miR‐125b‐5p attenuates EMT and CSC preferring via inhibiting STAT3. To date, few reports have investigated the relationship between miR‐125b‐5p and STAT3 in malignant tumors. Nevertheless, the function of miR‐125b‐5p is related to STAT3 in some benign cells. For instance, in Vγ2Vδ2 T cells from tuberculosis patients, an increase in miR‐125b‐5p is related to impairment of STAT3.^38^ In gangliocytes, miR‐125b‐5p regulation of STAT3 is related to a neuropathic pain phenotype.^39^ Interestingly, in the neural stem cell line C17.2, miR‐125b‐5p promotes cell differentiation into neurons by regulating STAT3 protein expression via binding to the first site in the *STAT3* 3′ UTR.^40^ This result suggests that STAT3 is associated with stemness, which is somewhat similar to our finding that the miR‐125b‐5p/STAT3 axis regulates the activity of HCC stem cells. STAT3 has been reported to be intimately associated with β‐Catenin‐mediated EMT potential and CSC activity in various malignancies.^41^, ^42^ These results raised our interest concerning whether the miR‐125b‐5p/STAT3 axis produced the EMT and CSC inhibition through β‐Catenin in hepatoma cells. Furthermore, we proved that activation of the miR‐125b‐5p/STAT3 axis promoted the phosphorylation and hydrolysis of β‐Catenin, resulting in suppression of wnt/β‐Catenin pathway activity, which ultimately produced the aforementioned therapeutic effects on hepatoma cells. The potential of miR‐125b‐5p as a therapeutic target for HCC has been fully demonstrated.

After the function and mechanism of the inhibition of hepatoma cells has been fully demonstrated, the best method to produce miR‐125b‐5p as a drug available for in vivo treatment becomes an important issue. In vivo delivery of naked miRNA is a challenge, because miRNAs are quickly degraded by nucleases and cleared via renal excretion. In addition, their hydrophilous characteristics and electronegativity prevent their ability to cross cell membranes.^18^ Traditionally, in vivo delivery systems for nucleic acids are classified as viral and nonviral vectors. Although viral vectors offer solutions for gene therapy, biosafety concerns have uncovered risks due to immune responses, insertional mutagenesis, generation of replication competent viruses, vector mobilization, and inadvertent transduction of nontarget cells.^43^ One approach used to overcome the predicament of miRNA therapy is based on nonviral nucleic acid nanocarriers.

Recently, polymeric carriers have become popular for in vivo nucleic acid delivery in an attempt to enhance cellular uptake and pharmacological effectiveness and reduce toxicity. Thus, these carriers have emerged as novel nanomedicine platforms for anticancer nucleic acids or contrast medium delivery.^43^, ^44^, ^45^, ^46^ Decoration with specific ligands or antibodies^47^ enables nanomedicines to recognize molecular signatures on the cell membrane for high efficiency in vivo recognition of cancer cells. Folate has been widely used as an ideal ligand for drug transmission nanocarriers to promote identification of HCC cells.^43^, ^44^, ^45^, ^46^, ^48^ We developed a folate‐conjugated nanocarrier (FaPPS) that achieved the in vivo delivery of therapeutic nucleic acid into hepatoma cells successfully in our previous study. Due to surface modification of folate, this nanocarrier has achieved efficient identification and gene transfer into hepatoma cells while avoiding transfection of normal cells and successfully reducing in vivo toxicity. At the same time, because it contains the sensitive MRI contrast agent SPION, the distribution process of this nanocarrier can be traced in vivo.^19^ In the present study, we utilized this nanocarrier for specific in vivo delivery of an miR‐125b‐5p plasmid to achieve inhibition of the EMT and CSC in hepatoma cells.

In the subcutaneous HCC model, we confirmed that the miR‐125b‐5p‐loaded nanomedicine enabled precise in vivo transfection of the miR‐125b‐5p plasmid and activation of the miR‐125b‐5p/STAT3 axis in hepatoma cells through RT‐PCR assay, western blot assay, and tumor growth curve assays. We also confirmed that this nanomedicine platform could achieve real‐time monitoring of therapeutic effects noninvasively in the treatment of cancer via the sensitive MRI imaging produced by SPION. This feature can help physicians easily observe the effects of gene therapy at any time and then individually adjust the treatment regimen. If the nanomedicine fails to produce the desired therapeutic effect (e.g., Figures 6F and 7A, Negative Control group), then doctors can replace another therapeutic gene in a timely manner (e.g., miR‐125b‐5p group). In the orthotopic HCC model, we used the aforementioned miR‐125b‐5p‐loaded nanomedicine to activate the miR‐125b‐5p/STAT3 axis of hepatoma cells and reduce wnt/β‐Catenin pathway activity, which realized the expected EMT and CSC in vivo inhibition functions.

## 4 Conclusions

In this study, clinical specimens were analyzed, and in vitro experiments were conducted to confirm that the miR‐125b‐5p/STAT3 axis could inhibit the invasiveness, migration, and stemness of HCC via the wnt/β‐Catenin pathway. Then, a folate‐conjugated nanocarrier loaded with an miR‐125b‐5p plasmid was designed as an intravenous genetic drug. After intravenous injection of the nanomedicine, high‐efficiency in vivo gene therapy and EMT/CSC inhibitory effects were achieved. The results suggest that we should develop an MRI‐visible nanomedicine platform that can achieve noninvasive therapeutic effect monitoring and timely individualized treatment course adjustment.

## 5 Experimental Section

Information regarding the following aspects of this study is available in the Supporting Information: patient studies, western blotting assay, RT‐PCR assay, plasmids, nanomedicine synthesis and characterization, cell culture, MTT assay, flow cytometry analysis, anchorage‐independent growth ability assay, colony formation assay, MRI scanning, liver orthotropic or subcutaneous tumors, blood concentrations analysis of nanomedicines, luciferase reporter assay, and statistical analysis.

## Conflict of Interest

The authors declare no conflict of interest.

## Supporting information

Supplementary

## References

[1] F. Bray , J. Ferlay , I. Soerjomataram , R. L. Siegel , L. A. Torre , A. Jemal , Ca‐Cancer J. Clin. 2018, 68, 394. 30207593 10.3322/caac.21492

[2] J. Bruix , M. Sherman , Hepatology 2011, 53, 1020. 21374666 10.1002/hep.24199 PMC3084991

[3] J. M. Llovet , M. Ducreux , R. Lencioni , A. M. Di Bisceglie , P. R. Galle , J. F. Dufour , T. F. Greten , E. Raymond , T. Roskams , T. De Baere , M. Ducreux , V. Mazzaferro , M. Bernardi , J. Hepatol. 2012, 56, S549.

[4] V. Mazzaferro , R. Lencioni , P. Majno , Semin. Liver Dis. 2014, 34, 415. 25369303 10.1055/s-0034-1394365

[5] M. Kudo , N. Izumi , N. Kokudo , O. Matsui , M. Sakamoto , O. Nakashima , M. Kojiro , M. Makuuchi , Dig. Dis. 2011, 29, 339. 21829027 10.1159/000327577

[6] M. Omata , L. A. Lesmana , R. Tateishi , P. J. Chen , S. M. Lin , H. Yoshida , M. Kudo , J. M. Lee , B. I. Choi , R. T. Poon , S. Shiina , A. L. Cheng , J. D. Jia , S. Obi , K. H. Han , W. Jafri , P. Chow , S. G. Lim , Y. K. Chawla , U. Budihusodo , R. A. Gani , C. R. Lesmana , T. A. Putranto , Y. F. Liaw , S. K. Sarin , Hepatol. Int. 2010, 4, 439. 20827404 10.1007/s12072-010-9165-7 PMC2900561

[7] J. Bruix , T. Takayama , V. Mazzaferro , G. Y. Chau , J. Yang , M. Kudo , J. Cai , R. T. Poon , K. H. Han , W. Y. Tak , H. C. Lee , T. Song , S. Roayaie , L. Bolondi , K. S. Lee , M. Makuuchi , F. Souza , M. A. Berre , G. Meinhardt , J. M. Llovet , Lancet Oncol. 2015, 16, 1344. 26361969 10.1016/S1470-2045(15)00198-9

[8] J. E. Zuckerman , M. E. Davis , Nat. Rev. Drug Dis. 2015, 14, 843. 10.1038/nrd4685 26567702

[9] B. P. Lewis , C. B. Burge , D. P. Bartel , Cell 2005, 120, 15. 15652477 10.1016/j.cell.2004.12.035

[10] N. Yang , N. R. Ekanem , C. A. Sakyi , S. D. Ray , Adv. Drug Delivery Rev. 2015, 81, 62. 10.1016/j.addr.2014.10.029 25450260

[11] A. Jayachandran , B. Dhungel , J. C. Steel , J. Hematol. Oncol. 2016, 9, 74. 27578206 10.1186/s13045-016-0307-9 PMC5006452

[12] M. Garofalo , C. M. Croce , Adv. Drug Delivery Rev. 2015, 81, 53. 10.1016/j.addr.2014.11.014 PMC4445133 25446141

[13] W. L. Hwang , J. K. Jiang , S. H. Yang , T. S. Huang , H. Y. Lan , H. W. Teng , C. Y. Yang , Y. P. Tsai , C. H. Lin , H. W. Wang , M. H. Yang , Nat. Cell Biol. 2014, 16, 268. 24561623 10.1038/ncb2910

[14] T. Shimagaki , T. Yoshizumi , N. Harimoto , S. Yoshio , Y. Naito , Y. Yamamoto , T. Ochiya , Y. Yoshida , T. Kanto , Y. Maehara , Hepatol. Res. 2018, 48, 313. 28984009 10.1111/hepr.12990

[15] B. G. Giray , G. Emekdas , S. Tezcan , M. Ulger , M. S. Serin , O. Sezgin , E. Altintas , E. N. Tiftik , Mol. Biol. Rep. 2014, 41, 4513. 24595450 10.1007/s11033-014-3322-3

[16] Y. Li , Y. Wang , H. Fan , Z. Zhang , N. Li , Biochem. Biophys. Res. Commun. 2018, 504, 277. 30177391 10.1016/j.bbrc.2018.08.172

[17] L. Hui , J. Zhang , X. Guo , Biomed. Pharmacother. 2018, 103, 1194. 29864898 10.1016/j.biopha.2018.04.098

[18] I. Fernandez‐Pineiro , I. Badiola , A. Sanchez , Biotechnol. Adv. 2017, 35, 350. 28286148 10.1016/j.biotechadv.2017.03.002

[19] Y. Guo , J. Wang , L. Zhang , S. Shen , R. Guo , Y. Yang , W. Chen , Y. Wang , G. Chen , X. Shuai , Hepatology 2016, 63, 1240. 26680504 10.1002/hep.28409

[20] J. M. Luna , J. M. Barajas , K. Y. Teng , H. L. Sun , M. J. Moore , C. M. Rice , R. B. Darnell , K. Ghoshal , Mol. Cell 2017, 67, 400. 28735896 10.1016/j.molcel.2017.06.025 PMC5603316

[21] T. Fang , H. Lv , G. Lv , T. Li , C. Wang , Q. Han , L. Yu , B. Su , L. Guo , S. Huang , D. Cao , L. Tang , S. Tang , M. Wu , W. Yang , H. Wang , Nat. Commun. 2018, 9, 191. 29335551 10.1038/s41467-017-02583-0 PMC5768693

[22] S. Ma , J. Sun , Y. Guo , P. Zhang , Y. Liu , D. Zheng , J. Shi , Theranostics 2017, 7, 3228. 28900506 10.7150/thno.19893 PMC5595128

[23] G. K. Scott , A. Goga , D. Bhaumik , C. E. Berger , C. S. Sullivan , C. C. Benz , J. Biol. Chem. 2007, 282, 1479. 17110380 10.1074/jbc.M609383200

[24] O. Govaere , J. Wouters , M. Petz , Y. P. Vandewynckel , K. Van den Eynde , A. Van den Broeck , S. Verhulst , L. Dolle , L. Gremeaux , A. Ceulemans , F. Nevens , L. A. van Grunsven , B. Topal , H. Vankelecom , G. Giannelli , H. Van Vlierberghe , W. Mikulits , M. Komuta , T. Roskams , J. Hepatol. 2016, 64, 609. 26592953 10.1016/j.jhep.2015.11.011

[25] H. R. Zhu , R. Z. Huang , X. N. Yu , X. Shi , E. Bilegsaikhan , H. Y. Guo , G. Q. Song , S. Q. Weng , L. Dong , H. Janssen , X. Z. Shen , J. M. Zhu , Tohoku J. Exp. Med. 2018, 245, 89. 29899182 10.1620/tjem.245.89

[26] L. L. Mei , W. J. Wang , Y. T. Qiu , X. F. Xie , J. Bai , Z. Z. Shi , PLoS One. 2017, 12, e185636. 10.1371/journal.pone.0185636 PMC5624607 28968424

[27] J. K. Kim , J. H. Noh , K. H. Jung , J. W. Eun , H. J. Bae , M. G. Kim , Y. G. Chang , Q. Shen , W. S. Park , J. Y. Lee , J. Borlak , S. W. Nam , Hepatology 2013, 57, 1055. 23079745 10.1002/hep.26101

[28] L. Zhao , W. Wang , Int. J. Clin. Exp. Med. 2015, 8, 18469. 26770454 PMC4694354

[29] Q. Bi , S. Tang , L. Xia , Du R , R. Fan , L. Gao , J. Jin , S. Liang , Z. Chen , G. Xu , Y. Nie , K. Wu , J. Liu , Y. Shi , J. Ding , D. Fan , PLoS One. 2012, 7, e40169. 22768249 10.1371/journal.pone.0040169 PMC3387011

[30] M. H. Hofmann , J. Heinrich , G. Radziwill , K. Moelling , Mol. Cancer Res. 2009, 7, 1635. 19825990 10.1158/1541-7786.MCR-09-0043

[31] S. Ibrahem , S. Al‐Ghamdi , K. Baloch , B. Muhammad , W. Fadhil , D. Jackson , A. S. Nateri , M. Ilyas , Int. J. Exp. Pathol. 2014, 95, 392. 25348333 10.1111/iep.12102 PMC4285465

[32] X. H. Wang , X. W. Meng , H. Xing , B. Qu , M. Z. Han , J. Chen , Y. J. Fan , C. Q. Lu , Z. W. Lu , Hepatogastroenterology 2011, 58, 487. 21661417

[33] D. Yang , M. Zhan , T. Chen , W. Chen , Y. Zhang , S. Xu , J. Yan , Q. Huang , J. Wang , Sci. Rep. 2017, 7, 43109. 28256505 10.1038/srep43109 PMC5335654

[34] E. Morelli , E. Leone , M. E. Cantafio , M. T. Di Martino , N. Amodio , L. Biamonte , A. Gulla , U. Foresta , M. R. Pitari , C. Botta , M. Rossi , A. Neri , N. C. Munshi , K. C. Anderson , P. Tagliaferri , P. Tassone , Leukemia 2015, 29, 2173. 25987254 10.1038/leu.2015.124 PMC4635336

[35] X. Yu , W. Shi , Y. Zhang , X. Wang , S. Sun , Z. Song , M. Liu , Q. Zeng , S. Cui , X. Qu , Sci. Rep. 2017, 7, 42226. 28176874 10.1038/srep42226 PMC5296742

[36] J. F. Zeng , Z. L. Zeng , K. Zhang , Y. Zhao , Y. M. Liu , J. J. Chen , H. Tong , D. H. Wei , Z. S. Jiang , Z. Wang , Cell Biol. Int. 2018, 42, 313. 29064597 10.1002/cbin.10896

[37] J. N. Zhou , Q. Zeng , H. Y. Wang , B. Zhang , S. T. Li , X. Nan , N. Cao , C. J. Fu , X. L. Yan , Y. L. Jia , J. X. Wang , A. H. Zhao , Z. W. Li , Y. H. Li , X. Y. Xie , X. M. Zhang , Y. Dong , Y. C. Xu , L. J. He , W. Yue , X. T. Pei , Hepatology 2015, 62, 801. 25953743 10.1002/hep.27887

[38] H. Shen , J. Gu , H. Xiao , S. Liang , E. Yang , R. Yang , D. Huang , C. Chen , F. Wang , L. Shen , Z. W. Chen , J. Infect. Dis. 2017, 215, 420. 27789724 10.1093/infdis/jiw511 PMC5853380

[39] K. K. Bali , M. Hackenberg , A. Lubin , R. Kuner , M. Devor , Molecular Pain. 2014, 10, 22. 24642266 10.1186/1744-8069-10-22 PMC4113183

[40] L. Xiu , Q. Xing , J. Mao , H. Sun , W. Teng , Z. Shan , Med. Sci. Monit. 2018, 24, 5041. 30027933 10.12659/MSM.907510 PMC6067029

[41] H. Huang , C. Wang , F. Liu , H. Z. Li , G. Peng , X. Gao , K. Q. Dong , H. R. Wang , D. P. Kong , M. Qu , L. H. Dai , K. J. Wang , Z. Zhou , J. Yang , Z. Y. Yang , Y. Q. Cheng , Q. Q. Tian , D. Liu , C. L. Xu , D. F. Xu , X. G. Cui , Y. H. Sun , Clin. Cancer Res. 2018, 24, 4612. 29691294 10.1158/1078-0432.CCR-18-0461

[42] Y. Gu , T. Chen , Z. Meng , Y. Gan , X. Xu , G. Lou , H. Li , X. Gan , H. Zhou , J. Tang , G. Xu , L. Huang , X. Zhang , Y. Fang , K. Wang , S. Zheng , W. Huang , R. Xu , Blood 2012, 120, 4829. 23074277 10.1182/blood-2012-06-434894 PMC4507036

[43] J. E. Vargas , L. Chicaybam , R. T. Stein , A. Tanuri , A. Delgado‐Canedo , M. H. Bonamino , J. Trans. Med. 2016, 14, 288. 10.1186/s12967-016-1047-x PMC5059932 27729044

[44] W. Zhuang , Y. Xu , G. Li , J. Hu , B. Ma , T. Yu , X. Su , Y. Wang , ACS Appl. Mater. Interfaces 2018, 10, 18489. 29737837 10.1021/acsami.8b02890

[45] Y. Cao , M. Liu , K. Zhang , G. Zu , Y. Kuang , X. Tong , D. Xiong , R. Pei , Biomacromolecules 2017, 18, 150. 28064499 10.1021/acs.biomac.6b01437

[46] R. Song , H. Song , Y. Liang , D. Yin , H. Zhang , T. Zheng , J. Wang , Z. Lu , X. Song , T. Pei , Y. Qin , Y. Li , C. Xie , B. Sun , H. Shi , S. Li , X. Meng , G. Yang , S. Pan , J. Zhu , S. Qi , H. Jiang , Z. Zhang , L. Liu , Hepatology 2014, 60, 1659. 25042864 10.1002/hep.27312

[47] Y. Guo , W. Chen , W. Wang , J. Shen , R. Guo , F. Gong , S. Lin , D. Cheng , G. Chen , X. Shuai , ACS Nano. 2012, 6, 10646. 23189971 10.1021/nn3037573

[48] K. S. Han , N. Li , P. A. Raven , L. Fazli , S. Ettinger , S. J. Hong , M. E. Gleave , A. I. So , Mol. Cancer Ther. 2015, 14, 1024. 25657336 10.1158/1535-7163.MCT-14-0771

