# Supplementary material for: Development of a Non‐Coding‐RNA‐based EMT/CSC Inhibitory Nanomedicine for In Vivo Treatment and Monitoring of HCC
Source: Adv Sci (Weinh). 2019 Mar 7;6(9):1801885. doi: 10.1002/advs.201801885 (PMC6498119; doi:10.1002/advs.201801885)
Supplement: Supplementary file 1 — Supplementary [file ADVS-6-1801885-s001.pdf]

Copyright WILEY-VCH Verlag GmbH & Co. KGaA, 69469 Weinheim, Germany, 2019.

Supporting Information

for *Adv. Sci.,* DOI: 10.1002/advs.201801885

Development of a Non-Coding-RNA-based EMT/CSC Inhibitory Nanomedicine for In Vivo Treatment and Monitoring of HCC

*Ruomi Guo, Zhiqiang Wu, Jing Wang, Qingling Li, Shunli Shen, Weiwei Wang, Luyao Zhou, Wei Wang, Zhong Cao, and Yu Guo**

Supplementary Materials

Supplementary Materials and Methods

Supplemental Table 1.

Information of Antibodies Used in this Study.

Antibodies	Company	Catalog No.	Application	Dilution fold
Anti-GAPDH	Abcam	ab8245	WB	1000
Anti-E-Cadherin	Abcam	ab15148	WB	200
Anti-alpha Catenin	Abcam	ab51032	WB	200
Anti-Vimentin	Abcam	ab92547	WB	250
Anti-Fibronectin	Abcam	ab2413	WB	1000
Anti-MMP2	Abcam	ab97779	WB	1000
Anti-MMP9	Abcam	ab38898	WB	1000
Anti-SNAIL	Abcam	ab82846	WB	500
Anti-SLUG	Abcam	ab27568	WB	1000
Anti-Nanog	Abcam	ab21624	WB	2000
Anti-Oct4	Abcam	ab181557	WB	1000
Anti-STAT3	Abcam	ab119352	WB,IHC	5000, 1600
Anti-E2F2	Abcam	ab138515	WB	2000
Anti-TNF alpha	Abcam	ab6671	WB	1000
Anti-SIRT1	Abcam	ab32441	WB	5000
Anti-TGF beta Receptor I	Abcam	ab31013	WB	1000
Anti-Smad4	Abcam	ab40759	WB	2000
Anti-SIRT7	Abcam	ab62748	WB	1000
Anti-MMP11	Abcam	ab53143	WB	1000
Anti-Raf1	Abcam	ab230850	WB	1000
Anti-beta Catenin	Abcam	ab32572	WB，IHC	5000, 250
Anti-beta Catenin (phospho S33 + S37)	Abcam	ab11350	WB	200

Anti-CD34	Abcam	ab81289	IF	100
Anti-CD133	Abcam	ab19898	IF，WB	1000, 250
Donkey F(ab')2 Anti-Rabbit IgG H&L (PE) preadsorbed	Abcam	ab7007	IF	250
Goat Anti-Rabbit IgG H&L (FITC)	Abcam	ab6717	IF	1000

Cell lines and culture

Cell lines Hep3B, PLC/PRF/5, HepG2 and SK-Hep1 were obtained from ATCC (Manassas, VA, USA). Cell lines HLE and HLF were obtained from the Japanese Collection of Research Bioresources Cell Bank (JCRB Cell Bank, Japan). Cell lines HCCLM3, SMMC-7721, HUH7, BEL7402 and L02 were obtained from Shanghai Cell Bank of Chinese Academy of Science (Shanghai, China). All cell lines used were authenticated. Human HCC cell lines HCCLM3, Hep3B, HepG2, SK-Hep1, HLE, HUH7, SMMC-7721, PLC/PRF/5, HLF, BEL7402, and human hepatocyte line LO2 were cultured in Dulbecco's Modified Eagle's Medium (DMEM), supplemented with 10% fetal bovine serum, 100 U/ml penicillin, and 100 μg/ml streptomycin. All of cell lines were cultured in a humidified chamber with 5% CO_2 at 37 °C. The involved cell lines were all purchased from the Shanghai Cell Collection (Shanghai, China). The cells were tested regularly for mycoplasma negative by MycoAlertTM Mycoplasma Detection Kit (Lonza, Basel, Switzerland).

Patients and specimens

A total of 64 HCC samples were histopathologically and clinically diagnosed by the Department of Pathology, First Affiliated Hospital of Sun Yat-Sen University from 2009 to 2013. HCC was diagnosed on the basis of CT, ultrasound or MRI characteristics and biochemistry (AFP serology and liver function enzymes), and was eventually confirmed by histopathology, according to the American Association for the Study of Liver Diseases guidelines.[1] All patients were older than 18 years of age with complete clinical and laboratory data. The inclusion criteria were that the samples contained matched tumors (percentage of tumor cells >70%) and corresponding normal liver tissue (>5 cm laterally from the edge of the cancerous region), the patient had a single primary lesion, and no adjuvant therapy. The patients who had the history of other solid tumors, had the secondary liver cancer from other primary regions, with mixed hepatocellular carcinoma and cholangiocarcinoma were excluded from this study. Patients were regularly followed up at the outpatient clinics every 3 months for the first 2 years, every 6 months for the next 3 years, and once

annually thereafter. Patients with no follow-up data were excluded. Fresh primary human HCC tumor (T) tissues and corresponding adjacent nontumor (ANT) liver tissues were acquired from the surgical specimen archives of the First Affiliated Hospital of Sun Yat-Sen University, Guangzhou, China. For the use of these clinical materials and data for research purposes, prior patients' written consents and approval from the Institutional Research Ethics Committee (IREC) of the First Affiliated Hospital of Sun Yat-Sen University were obtained, in accordance with rules and regulations concerning ethical issues on research use of human subjects in China. The study protocol conformed to the ethical guidelines of the current Declaration of Helsinki was approved by the IREC of the First Affiliated Hospital of Sun Yat-Sen University.

Real-time quantitative RT-PCR (qPCR)

Total RNA was extracted and purified using TRIzol reagents (Invitrogen, Carlsbad, CA) and RNeasy mini kit (Qiagen Inc, Valencia, CA) according to the manufacturer's instructions. The relative mRNA levels were determined by reverse transcription and qPCR with Sybr Green dye (Invitrogen) as described previously.[2] For miRNA analysis, cDNAs were synthesized using the PrimeScript RT reagent kit (Takara). Then, qPCRs were performed by using the Takara SYBR Premix Ex Taq II (Takara) and the 7500 Real-Time PCR System (Applied Biosystems). Quantitative real-time PCR (qRT-PCR) analysis of miR-125b-5p was performed by using a commercially purchased kit specifically designed for quantifying this miRNA (TaqMan MicroRNA Assay Kit, Applied Biosystems, Foster City, CA). The expression of miRNA was defined based on the threshold cycle (Ct), and relative expression levels were calculated as $2^{-[(\text{Ct of miR-125b-5p}) - (\text{Ct of U6})]}$ after normalization with reference to expression of U6 small nuclear RNA.

Western blotting analysis

Three groups of HCCLM3 and HUH7 were harvested 24 h after transfections respectively. Equal amounts of protein lysates (30 μg) were separated by 10 %

SDS-PAGE for immunoblots with primary antibodies. A horseradish peroxidase-conjugated anti-rabbit or anti-mouse immunoglobulin-G antibody was used as the secondary antibody. Signals were detected using enhanced chemiluminescence reagents (Amersham Biosciences). All the experiments were independently performed three times as the least. Information and dilution of antibodies used in this study were listed in Supplementary Table 1.

Plasmids and stable cell lines

The pre-miR-125b-1 was synthesized by Invitrogen and ligated into the EcoR I/ Bgl II sites of the pMSCV-neo vector. For establishment of miR-125b-5p overexpression stable cell lines, pMSCV-neo-miR-125b-1 plus with pVPack-GP and pVpack-VSV-G were co-transfected into 293T cells to package viral particles. The viruses were collected and infected HCCLM3 and HUH7 cells. 48 h after infection, the cells were selected with G418-containing (500 μg/ml) medium for at least 10 days. Then the expression of miR-125b-5p was detected by quantitative real-time PCR. The expression of miR-125b-5p was defined based on the threshold cycle (Ct), and relative expression levels were calculated as $2^{-[(\text{Ct of miR-125b-5p}) - (\text{Ct of U6})]}$ after normalization with reference to expression of U6 small nuclear RNA. After validating the successful overexpressing miR-125b-5p, the cells were maintained in a low concentration of G418 (200 μg/ml) containing medium and the expression of miR-125b-5p was assessed monthly.

Wound-healing assays

For wound-healing assays, transfected cells were plated in six-well plates, and cells were maintained in serum-free medium. When the HCCLM3 and HUH7 including miR-125b-5p group, negative control group and blank control group confluence reached 80–90%, a scratch was made using a 10-μl pipette tip, after serum starved for 24 h. The wounded monolayer was washed with PBS and imaged 48 h after scratching using an Olympus camera system. Wound healing assays were conducted in triplicate.

Cell invasion assay

For invasion assays, three groups of HCCLM3 and HUH7 were resuspended in 200 μl of serum-free DMEM medium and seeded onto Boyden chambers (Corning, NY, USA) coated with Matrigel. The chambers were then incubated in DMEM with 20% FBS at 37 °C in 5% CO_2. After 24 h, the cells adhering to the chamber's lower surface were fixed, whereas cells remaining on the upper surface were removed. After staining in a dye solution containing 0.05% crystal violet, the cells from three randomly selected high power fields were counted under a microscope (Olympus, Tokyo, Japan). Invasion assays were conducted in triplicate.

Tube formation assay

The HUVEC cells commercially obtained from Vec Technologies (Rensselaer, NY), and the tube formation assay was carried out as described.[3] In brief, 96-well plates were coated with 50 μl Matrigel. 2×10^4 HUVEC cells were added to each well and incubated respectively in medium preconditioned with three groups of HCCLM3 and HUH7 cells at 37°C for 6–8 h. Images were acquired under an inverted microscope. Angiogenesis potential was quantified by measuring the length of tube walls formed between discrete endothelial cells in each well relative to the control. Tube formation assays were conducted in triplicate.

Flow cytometry

Three groups of HCCLM3 and HUH7 were digested using trypsin and resuspended at 1×10^6 cells per mL in DMEM that contained 2% FBS and pre-incubated at 37°C for 30 min with or without 100 μM verapamil (Sigma-Aldrich, 1711202) to inhibit ABC transporters. Cells were subsequently incubated for 90 min at 37°C with 5 μg/ml Hoechst 33342 (Sigma-Aldrich B2261). Then, cells were incubated on ice for 10 min and washed with ice-cold PBS before flow cytometry analysis. Data were analyzed using Summit 5.2 software (Beckman Coulter).

Sphere formation assay

Various cells (1000 cells per well) were plated onto a 24-well ultra-low attachment plate (Corning) in serum free DMEM/F12 medium supplemented with N2 plus media supplement (Invitrogen), 20 ng/ml epidermal growth factor (Invitrogen), 20 ng/ml basic fibroblast growth factor (Invitrogen) and 4 mg/ml heparin (Sigma-Aldrich). After 10 days of culture, the number of spheres larger than 50 μm was counted under an inverted microscope. The spheres were photographed under a microscope and the diameters of spheres were measured. After counting sphere numbers, all tumor spheres were collected by spin down and trypsinized into single cells. The total cell number divided by the sphere number is the average cell number of each sphere. Sphere formation assays were conducted in triplicate.

Vectors construction

The 3' untranslated region (3'UTR) of STAT3 was amplified and subcloned into the pMIR-Report luciferase vector (Ambion, Austin, TX, USA). Mutants (MUT) of the seed region of the putative miRNA-binding sites in 3'UTR were generated by point mutation PCR and used in luciferase reporter assay. The sequence of STAT3 were obtained by PCR (Origene, Rockville, MD, USA) for rescue experiment and subcloned into the pCMV-NFlag vector (Beyotime Biotechnology, Jiangsu, China) to construct miR-125b-5p eukaryotic expression plasmid.

Luciferase reporter assay

HCCLM3 and HUH7 cells were seeded respectively in 96-well plates at a density of 5000 cells per well. After 24 h, the psiCheck2 vectors that carrying 3'UTR DNA fragment containing the indicated miR-125b-5p binding site or with the target sequence mutated were transfected into cells with Lipofectamine 2000 (Invitrogen). Twenty four hours later, the miR-125b-5p mimics were transfected into the cells. Another 48 h later, cells were collected and suspended in lysis buffer (Promega, Madison, WI, USA), and luciferase activities were monitored for luciferase and renilla activity using reagents in dual-luciferase reporter assay kit and a luminometer

(Promega), as recommended by the manufacturer.

To investigate the transcriptional activity of β-catenin, TOP/FOP flash assay was performed. Different groups of HCCLM3 and HUH7 cells were transfected with TOP or FOP flash plasmids along with SV40-Renilla plasmid (control for transfection) using Lipofectamine 2000 (Invitrogen). On the following day, we transferred the cells with fresh media. 48 h after transfection, luciferase activity were detected according to the manufacturer's protocol (Promega). All experiments were performed for at least three times in triplicate, and the data of each group are presented as mean ± standard deviation (SD).

Coimmunoprecipitation analysis.

HCCLM3 and HUH7 cells were transfected with flag-STAT3 vector and HA-β-catenin vector using Lipofectamine 2000. After 48 h, cells were lysed in lysis buffer (1% NP-40, 25 nM HEPES, 150 mM NaCl, 1 mM EDTA, 5% Glycerol, pH 7.4). STAT3 was immunoprecipitated using anti-FLAG Agarose overnight at 4 °C, and β-catenin was immunoprecipitated using anti-HA Agarose. The immunoprecipitated material was eluted (Sigma-Aldrich) and analyzed by western blotting assay.

Synthesis and characterization of delivery agents

The Folate-conjugated block copolymer, Fa-PEG-g-PEI, was synthesized according to the previously described method.[4] Briefly, HO-PEG-NH_2 [5] and folic acid (1 g) were dissolved in anhydrous DMSO (20 mL). N-hydroxysuccinimide (NHS, 0.9 g) and dicyclohexylcarbodiimide (DCC, 0.5 g) were added and the mixture was stirred overnight at room temperature. Under an argon atmosphere, the mixture was filtrated through the 0.22 μm syringe filter to remove 1,3-dicyclohexyl urea (DCU), and then the filtrate was mixed with a DMSO solution of HO-PEG-NH_2(0.53g) and TEA (pH 8.0). The mixture was allowed to react for 24 h at room temperature, dialyzed against deionized water (MWCO: 1000 Da), and lyophilized. Fa-PEG-OH thus prepared was converted into Fa-PEG-COOH by reaction with succinic anhydride

(SA). Fa-PEG-OH and SA in a 1:5 molar ratio were dissolved in 20 mL anhydrous chloroform and refluxed at 70°C for 48 h. After chloroform was removed by distillation, polymer was re-dissolved in 20 mL deionized water and dialyzed against water for two days to remove succinic acid and succinic anhydride. Polymer solution was freeze-dried to obtain Fa-PEG-COOH. Finally, hyperbranched PEI 25 kDa, Fa-PEG-COOH and NHS were dissolved in DMSO and magnetically stirred for 24 h at room temperature to produce Fa-PEG-g-PEI. The solution was dialyzed (MWCO: 14000 Da) in distilled water for 3 day and then lyophilized. Nontargeted mPEG-g-PEI was synthesized using the same approach.

Preparation of magnetic nanocarriers (Fa-PEG-g-PEI-SPION)

SPION was complexed with Fa-PEG-g-PEI by the ligand exchange method as reported.[6] In brief, Fa-PEG-g-PEI (200 mg) was dissolved in 2 mL of chloroform, and SPION (10 mg) was dispersed into the solution. The reaction mixture was stirred overnight at room temperature before precipitating into large amount of hexane. The precipitate was collected by centrifugation, washed twice with hexane and vacuum-dried. The product thus prepared was dispersed into double distilled water under sonication and filtered through a 220 nm membrane to remove large aggregates.

Nanomedicine formation.

miR-125b-5p or negative control-miR plasmid (4 mg) and an appropriate amount of nanocarrier were dissolved separately in 0.9% sodium chloride solution. The two solutions were mixed by vigorous pipetting and then kept at room temperature for 60 min to allow polyplex formation. The amount of delivery agent (Fa-PEG-g-PEI-SPION) to complex plasmid was based on the designed experimental N/P ratio (10), which was calculated as the number of nitrogen atoms in delivery agents over that of the phosphate groups in plasmids.

In vitro and vivo MRI

The HCCLM3 or HUH7 cells cultured in vitro were imaged using a clinical 1.5-T

MRI system (Achieva; Philips Medical Systems, Best, the Netherlands) with an 11-cm circular coil. Fast spin echo (FSE) T2-weighted images were acquired using the following parameters: TR/TE = 500/15 ms, number of signal acquisitions (NSA) = 2; TR/TE = 2600/100 ms, NSA = 4; FOV = 70–80 mm, matrix = 256 × 256, section thickness = 1.5 mm.

In vivo MRI was performed after tail vein injection of nanomedicines for 2 hours in each group to detect the size and signal of the tumour using a clinical 1.5-T system (Achieva; Philips Medical Systems) with a 50× 69 mm linearly polarised birdcage radio frequency rat coil (Shanghai Chenguang Medical Technologies, China). Axial liver images were obtained using T2-weighted imaging (TR/TE = 2600/100 ms; NSA = 3); FOV = 70 mm, matrix = 256 × 256, and section thickness = 1.0 mm.

Fluorescence study on in vitro cellular

To enable confocal laser scanning microscopic (CLSM) observation of the cells after incubation, the nanocarrier Fa-PEG-g-PEI-SPION and pDNA were labeled with Oregon Green 488 and POPO-3, respectively, according to the manufacturer's protocols.[7] HCCLM3 or HUH7 cells were seeded at a density of 5×10^5 cells per well in 6-well plates. The POPO-3 labeled pDNA was complexed with the Oregon Green-labeled nanocarrier at N/P=10. The formed polyplex (at Fe concentrations of 20 μg/mL) was then added into culture medium. After 0.5 h, cells were washed 3 times with fresh PBS free of polyplex, fixed and further incubated by the nucleus DNA-staining agent DAPI. CLSM observation was carried out using a Zeiss LSM 510 META microscope (Carl Zeiss Co., Ltd., Gottingen, Germany).

Animal experiment

Male BALB/c nude mice (4-5 weeks old, 18-20 g) were purchased from Vital River Laboratories (Beijing, China) and were housed in a barrier facility on a 12 h light/dark cycle in the Center for Experimental Animals of Sun Yat-Sen University. All studies involving animals were approved by the Institutional Animal Care and Use Committee of the First Affiliated Hospital of Sun Yat-Sen University. All

experimental procedures involving animals were in accordance with the Guide for the Care and Use of Laboratory Animals (NIH publication nos. 80-23, revised 1996) and were performed according to the institutional ethical guidelines for animal experiments. The retention and use of animals were in compliance with national and local laws and regulations. HCCLM3 or HUH7 cells (1×10^7 cells in final concentration 25% Matrigel) were inoculated into the lateral skin of right buttocks, or right lobe of liver, of BALB/c mice to establish subcutaneous HCC model or orthotopic liver cancer model. Mice after tumor cell injection were randomly divided into three groups for each model: Blank Control group, Negative Control group, or miR-125b-5p group (12 for each group). In Negative Control group or miR-125b-5p group, nanomedicines contain 8 μg plasmids were continuous tail vain injected once every 3 days and observation for 30 days. The blank control group injected the same volume of PBS. Subcutaneous tumor volumes were measured using external caliper or MRI T2 scanning and calculated using the following equation: $(L\times W^2)/2$. Mice were sacrificed 30 days after inoculation and the tumors were excised and subjected to pathologic examination.

Blood concentrations of nanomedicines.

The BALB/c mice were randomly divided into three groups (n = 6). The Alexa Fluor 750-labeled FaPPS containing miR-125b-5p, Alexa Fluor 750-labeled FaPPS containing Negative Control miR or PBS were tail vein injected into the mice of different groups, respectively. The blood samples (150 μL each) were obtained every 3 h after injection. To analyze the blood concentrations of nanomedicines, the fluorescence intensity of Alexa Fluor 750 in the blood was detected by fluorescence spectrophotometer after a 20 fold dilution (Ex: 750 nm; Em: 778 nm). The fluorescence intensities of blood samples at 0 h after injection were employed for data normalization, and the Blank Control mice receiving PBS were analyzed for data calibration.[8, 9]

Prussian blue staining

Prussian blue staining was performed on the tumor tissues from each animal to identify the presence of ferric iron (Fe). The slides were thawed and hydrated in distilled water for 2 minutes, followed by transfer to 1:1 solution of 10% potassium ferrocyanide (Sigma-Aldrich) and 20% HCl for 20 minutes. After rinsing with distilled water, the slides were counterstained by nuclear-fast red (Sigma-Aldrich) solution for 5 minutes. Slides were then washed in distilled water for 5 minutes and dehydrated in increasing concentrations of ethanol (70%, 80%, 90%, 95%, and 100%) followed by xylene (2× 3 minutes), and afterwards coverslipped with PermountTM (Sigma-Aldrich). Tumor tissues after Prussian blue staining were observed using an Olympus BX40 microscope with a SPOT RT3 digital camera (Diagnostic Instruments Inc., Stirling Heights, MI, USA) under bright field illumination.

Immunofluorescence assay and Immunohistochemistry assay

Tumor samples from HCCLM3 or HUH7 cells were fixed 10% buffered formalin and embedded in paraffin. 4 μm thick tissue sections were cut. All sections were deparaffinnized and dehydrated with graded alcohol. The sections were then washed 10 min in phosphate-buffered saline (PBS) (pH 7.2). The endogenous peroxidase activity was quenched by incubation in methanol containing 3% H_2O_2 for 10 min at room temperature, then heated for 30 min at 95°C to repair antigens and finally rinsed in PBS. After several washes in PBS, the sections were blocked with goat serum for 20 min at room temperature, and then incubated with CD133 or CD34 primary antibodies overnight at 4°C in a humidified chamber. Replacing the primary antibodies by PBS, negative control staining was using performed, the slides were treated with polymer enhancer (Reagent A) for 20 min at room temperature. After a complete wash in PBS, the slides were treated with fluorescent secondary antibodies for 30 min at room temperature. After a complete wash in PBS, the slides were develop in freshly prepared diaminobenzedine solution (DAB) for 8 min, and then counterstained with hematoxylin, dehydrated, airdried, and mounted.

In addition, some slides of HCCLM3 or HUH7 tumors were pretreated with an antigen retrieval method by heating the slides in an autoclave in citrate buffer (10 mM,

pH 6.0) for 90 seconds except those stained for STAT3 and β-catenin. EDTA-Tris buffer (1 mM, pH 9.0) was used for pretreating before staining. After rinsing in TBS (pH7.6), the specimens were incubated for 2 h at 37°C with anti-STAT3 or anti-β-catenin antibody. Subsequently, all slides were incubated with Envision HRP antibody working fluid (Dako Company) for 30 minutes at 37°C, and then developed with DAB-H_2O_2 solution (Dako Company). The cell nuclei were stained with Meyer's hemotoxylin.

Statistical analysis

The data are expressed as the means ± standard deviation (S.D.). Experimental data were analyzed by Student's t-tests for two groups and two-way analysis of variance for multiple groups. All statistical tests were performed using SPSS 16.0 software (SPSS, Inc., Chicago, IL), and statistical significance was set at $P < 0.01$.

References:

[1] J. Bruix, M. Sherman, Hepatology. **2005**, 42, 1208.

[2] A. Kawaguchi, N. Yajima, Y. Komohara, H. Aoki, N. Tsuchiya, J. Homma, M. Sano, M. Natsumeda, T. Uzuka, A. Saitoh, H. Takahashi, Y. Sakai, H. Takahashi, Y. Fujii, T. Kakuma, R. Yamanaka, Int. J. Oncol. **2012**, 40, 721.

[3] G. Z. Yu, S. Reilly, A. J. Lewandowski, C. Aye, L. J. Simpson, L. Newton, E. F. Davis, S. J. Zhu, W. R. Fox, A. Goel, H. Watkins, K. M. Channon, S. M. Watt, T. Kyriakou, P. Leeson, Hypertension. **2018**, 72, 937.

[4] B. Liang, M. L. He, C. Y. Chan, Y. C. Chen, X. P. Li, Y. Li, D. Zheng, M. C. Lin, H. F. Kung, X. T. Shuai, Y. Peng, Biomaterials. **2009**, 30, 4014.

[5] S. Cammas, Y. Nagasaki, K. Kataoka, Bioconjug Chem. **1995**, 6, 226.

[6] Y. Guo, J. Wang, L. Zhang, S. Shen, R. Guo, Y. Yang, W. Chen, Y. Wang, G. Chen, X. Shuai, Hepatology. **2016**, 63, 1240.

[7] Y. Guo, W. Chen, W. Wang, J. Shen, R. Guo, F. Gong, S. Lin, D. Cheng, G. Chen, X. Shuai, Acs Nano. **2012**, 6, 10646.

[8] J. Li, X. Yu, Y. Wang, Y. Yuan, H. Xiao, D. Cheng, X. Shuai, Adv. Mater. **2014**, 26, 8217.

[9] Y. Guo, Z. Wu, S. Shen, R. Guo, J. Wang, W. Wang, K. Zhao, M. Kuang, X. Shuai, Nat Commun. **2018**, 9, 3430.

Supplementary Figures and Figure Legends

Supplementary Figure 1

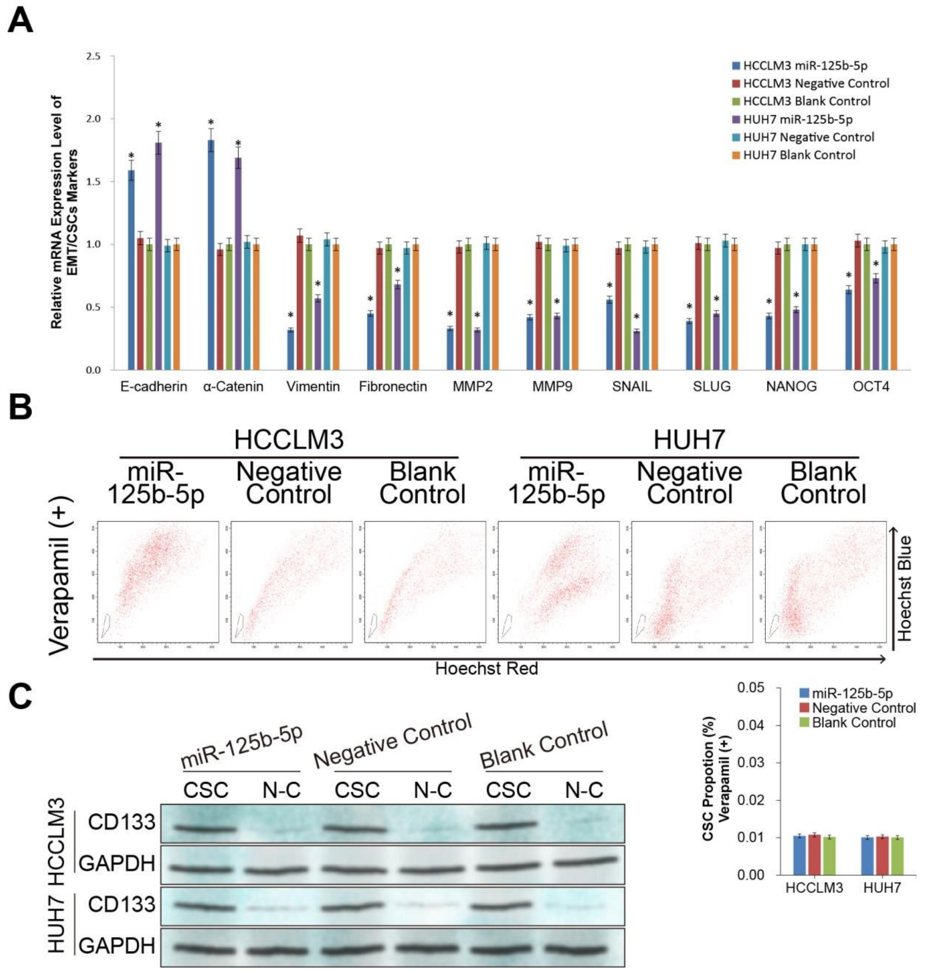

Supplementary Figure 1. (A) The relative mRNA levels of epithelial markers (E-cadherin and α-Catenin), mesenchymal markers (Vimentin, Fibronectin, MMP2, MMP9, SNAIL and SLUG) and cancer stem cells (CSC) markers (NANOG and OCT4) in the indicated groups of HCCLM3 and HUH7 cells. The expression levels were normalized to the expression level of the non-transfected Blank Control cells. See also Figure 2B. (B) CSC were detected by the flow cytometry side population cell assay with the presence of verapamil in HCCLM3 and HUH7 cells. (C) Western blotting analysis of the expression of CD133 in the sorted CSC and non-CSC (N-C) of each group. All experiments were performed in triplicate, and the data from each group are presented as the mean±SD. n=3, * P<0.01.

Supplementary Figure 2

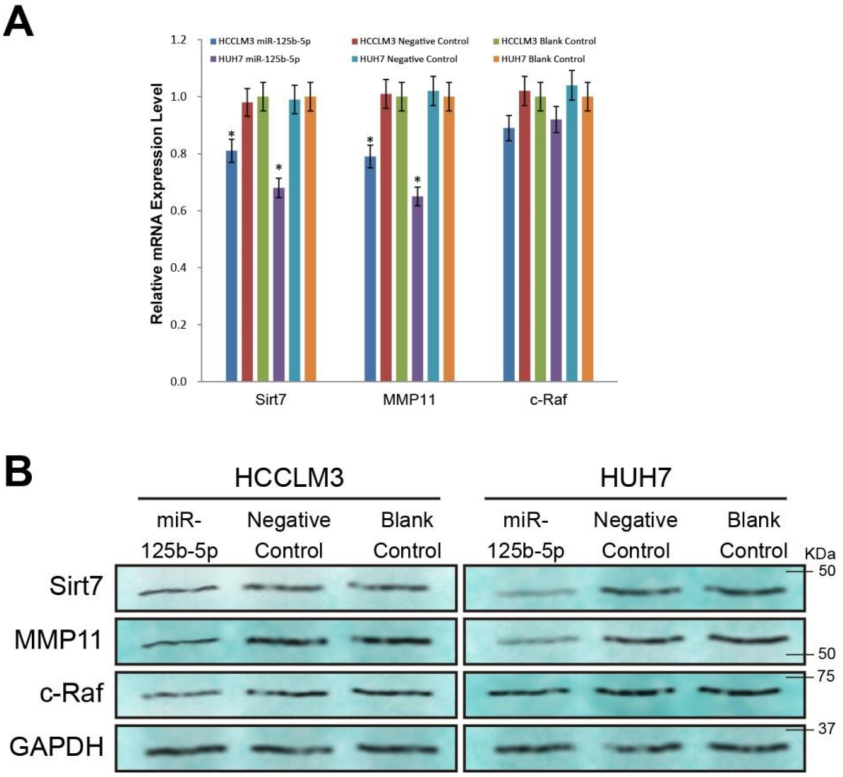

Supplementary Figure 2. Relative mRNA expression A) and protein level B) of Sirt7, MMP-11 and c-Raf in the indicated groups of cells. The expression levels were normalized to the expression level of the non-transfected Blank Control cells. All experiments were performed in triplicate, and the data from each group are presented as the mean±SD. n=3, * P<0.01.

Supplementary Figure 3

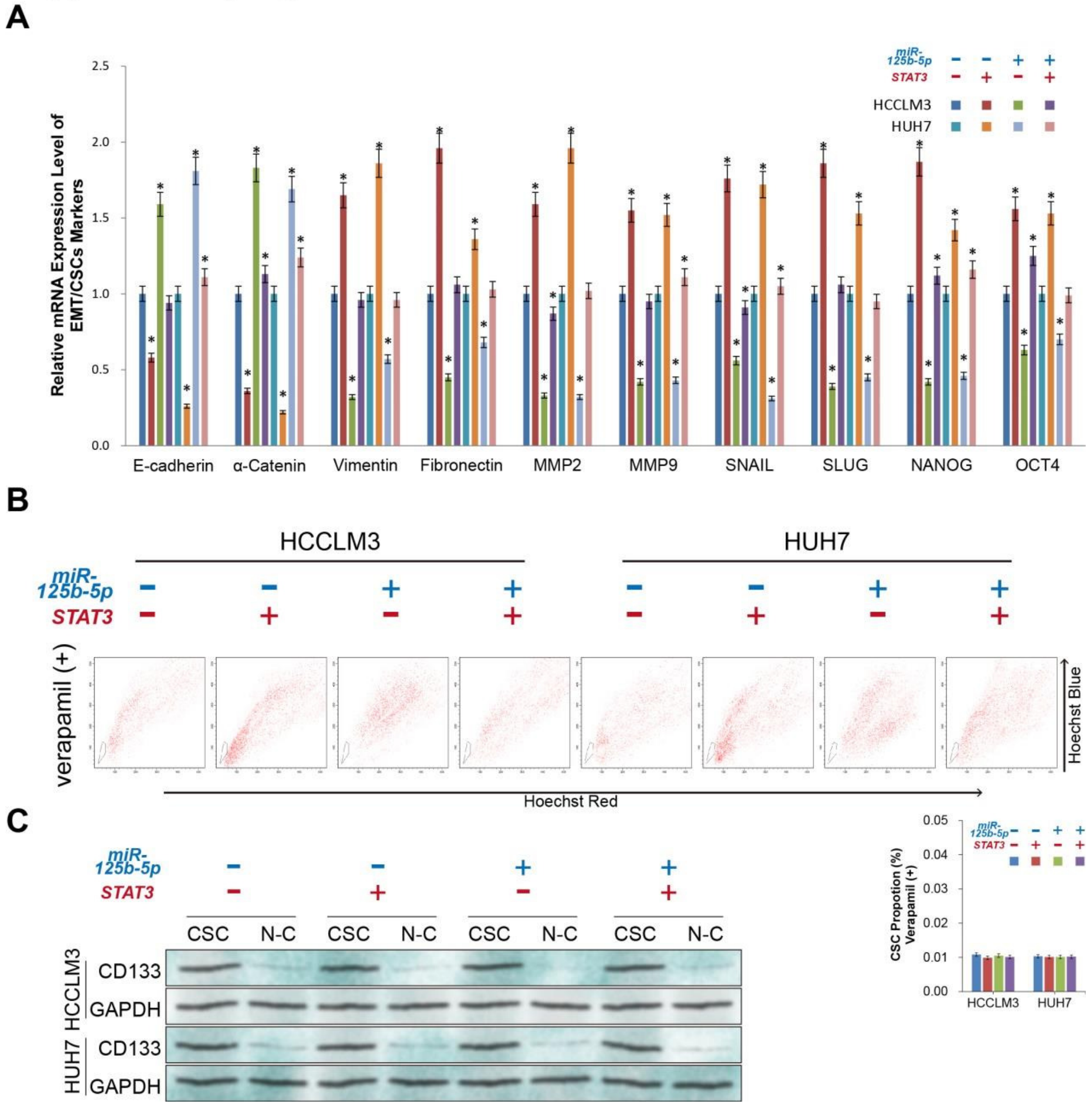

Supplementary Figure 3. A) The relative mRNA levels of epithelial markers (E-cadherin and α-Catenin), mesenchymal markers (Vimentin, Fibronectin, MMP2, MMP9, SNAIL and SLUG) and cancer stem cells (CSC) markers (NANOG and OCT4) in the indicated groups of cells. The expression levels were normalized to the expression level of the non-transfected miR-125b-5p (-)/STAT3 (-) cells. See also Figure 4B. B) CSC were detected by the flow cytometry side population cell assay with the presence of verapamil in HCCLM3 and HUH7 cells. C) Western blotting analysis of the expression of CD133 in the sorted CSC and non-CSC (N-C) of each group. All experiments were performed in triplicate, and the data from each group are presented as the mean±SD. n=3, * P<0.01.

Supplementary Figure 4

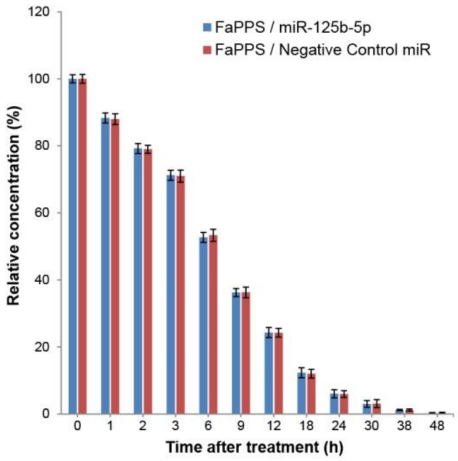

Supplementary Figure 4. In vivo dosage levels and duration of the Folate-PEG-g-PEI-SPION (FaPPS) nanomedicines measured by the fluorescence intensity of the blood collected after single tail-vein injection. The data from each group are presented as the mean±SD, n=12.
